# Low-Frequency Mutational Heterogeneity of Invasive Ductal Carcinoma Subtypes: Information to Direct Precision Oncology

**DOI:** 10.3390/ijms20051011

**Published:** 2019-02-26

**Authors:** Meagan B. Myers, Karen L. McKim, Malathi Banda, Nysia I. George, Barbara L. Parsons

**Affiliations:** 1 Division of Genetic and Molecular Toxicology, National Center for Toxicological Research, U.S. Food and Drug Administration, Jefferson, AR 72079, USA; karen.mckim@fda.hhs.gov (K.L.M.); malathi.banda@covance.com (M.B.); barbara.parsons@fda.hhs.gov (B.L.P.); 2 Division of Bioinformatics and Biostatistics, National Center for Toxicological Research, U.S. Food and Drug Administration, Jefferson, AR 72079, USA; nysiainet@gmail.com

**Keywords:** invasive ductal carcinoma, breast cancer, mutation, cancer-driver, triple-negative breast cancer, TNBC, heterogeneity, PIK3CA, subclonal

## Abstract

Information regarding the role of low-frequency hotspot cancer-driver mutations (CDMs) in breast carcinogenesis and therapeutic response is limited. Using the sensitive and quantitative Allele-specific Competitor Blocker PCR (ACB-PCR) approach, mutant fractions (MFs) of six CDMs (*PIK3CA* H1047R and E545K, *KRAS* G12D and G12V, *HRAS* G12D, and *BRAF* V600E) were quantified in invasive ductal carcinomas (IDCs; including ~20 samples per subtype). Measurable levels (i.e., ≥ 1 × 10^−5^, the lowest ACB-PCR standard employed) of the *PIK3CA* H1047R, *PIK3CA* E545K, *KRAS* G12D, *KRAS* G12V, *HRAS* G12D, and *BRAF* V600E mutations were observed in 34/81 (42%), 29/81 (36%), 51/81 (63%), 9/81 (11%), 70/81 (86%), and 48/81 (59%) of IDCs, respectively. Correlation analysis using available clinicopathological information revealed that *PIK3CA* H1047R and *BRAF* V600E MFs correlate positively with maximum tumor dimension. Analysis of IDC subtypes revealed minor mutant subpopulations of critical genes in the MAP kinase pathway (*KRAS*, *HRAS*, and *BRAF*) were prevalent across IDC subtypes. Few triple-negative breast cancers (TNBCs) had appreciable levels of *PIK3CA* mutation, suggesting that individuals with TNBC may be less responsive to inhibitors of the PI3K/AKT/mTOR pathway. These results suggest that low-frequency hotspot CDMs contribute significantly to the intertumoral and intratumoral genetic heterogeneity of IDCs, which has the potential to impact precision oncology approaches.

## 1. Introduction

Breast cancer is a heterogeneous disease, presenting with a spectrum of clinical features that impact prognosis and clinical response to treatment. Classically, breast tumors are characterized by the presence or absence of a hormone receptor [HR; estrogen receptor (ER) or progesterone receptor (PR)) and/or the overexpression of human epidermal growth factor receptor-2 (HER2)]. Breast tumors that are HR+ and/or HER2+ account for 80–85% of breast cancer cases. The remaining 15–20% of breast cancer cases are classified as triple-negative breast cancer (TNBC), which are defined by the lack of ER, PR, and HER2 overexpression. It is now standard care to evaluate new breast cancers for the presence of these markers [1,2,3].

Intratumoral heterogeneity (i.e., cells or subclones of cells with differing genetic, epigenetic and/or phenotypic characteristics) may have a significant impact on the clinical management of breast cancer, particularly with regard to diagnosis, prognosis, and the efficacy of molecularly targeted therapies and chemotherapies. In recent years, it has become clear that intertumoral and intratumoral genetic heterogeneity poses a significant obstacle in the development of durable therapeutics and treatment strategies for breast cancer [4,5]. Subclones of cells with specific cancer-driver gene mutations (i.e., mutations within genes that confer a selective growth advantage, resulting in cancer; CDMs) may confer a sensitivity or resistance to a targeted therapy or be more likely to metastasize. Thus, accurate characterization of subclonal CDMs by highly sensitive and quantitative methodologies is informative and may have implications for the clinical management of disease.

Large-scale genomic analyses of breast cancer (mostly utilizing next generation sequencing and array technologies) have established the spectrum of high-frequency/predominant somatic gene mutations in various histological types of breast cancer, but at low prevalence. In breast carcinomas, the two most frequently mutated genes, *TP53* and *PIK3CA*, are present in 2937/5906 (49.7%) and 4479/16715 (26.8%) of solid tumors, respectively (COSMIC database; [6]), garnering interest as potential avenues for targeted therapy. Progress in finding other somatic gene mutations as actionable targets for therapy has been largely disappointing. According to the COSMIC database, 13 out of the 20 top genes mutated in breast carcinomas are present in < 5% of the samples analyzed [6]. However, this may be attributed to the heterogeneous nature of breast cancer and the use of genome analyses with relatively low sensitivities.

Indeed, when utilizing more sensitive mutation detection techniques, low-frequency hotspot CDMs are present in most, if not all, ductal carcinomas, as well as normal breast tissue [7]. Allele-specific Competitor Blocker PCR (ACB-PCR) employs a priming strategy that enables the quantification of specific hotspot point mutations, such as hotspot point mutations found in cancer-driver genes, down to three mutant molecules in a background of 300,000 wild-type molecules [8]. ACB-PCR is a highly sensitive and quantitative mutation detection method. Direct comparisons with other methods (Diacarta’s QClamp^®^, *PIK3CA* H1047R allele-specific PCR with a sensitivity of 1 × 10^−3^ [7]; Droplet Digital PCR, *PIK3CA* H1047R, sensitivity of 1.7–2.4 × 10^−4^ [9]; and error-corrected next-generation sequencing, sensitivity of 1 × 10^−4^ [10]) demonstrate relatively good concordance with ACB-PCR within their measurable ranges, but with ACB-PCR detecting additional, lower-frequency mutations. Using ACB-PCR, it was discovered that hotspot CDMs are prevalent in normal human tissues. Indeed, the inter-individual variation of hotspot CDMs is correlated with the known tissue specific importance of the different mutations as drivers within different human tissues [11], suggesting that ACB-PCR was able to describe the stochastic, early clonal expansion of CDMs in normal tissues. Several studies have confirmed that CDMs are present and measurable in a variety of normal human tissues, provided a sufficiently sensitive mutation detection method is employed [12,13,14,15,16,17,18]. To examine the potential impact of low-frequency CDMs in breast carcinogenesis and targeted therapeutics, in previous work, we used ACB-PCR to quantify five hotspot CDMs (*PIK3CA* H1047R, *KRAS* G12D, *KRAS* G12V, *HRAS* G12D, and *BRAF* V600E) in human breast invasive ductal carcinomas (IDCs; [7]), and six hotspot CDMs (*PIK3CA* H1047R, *PIK3CA* E545K, *KRAS* G12D, *KRAS* G12V, *HRAS* G12D, and *BRAF* V600E) in the normal human breast [7,11]. These targets were selected in part because they contain mutational hotspots (i.e., large percentages of the mutations within these genes occur within one or a few specific codons), making them ideal targets for the ACB-PCR methodology, which interrogates only a single base substitution in each assay. In addition, these CDMs were chosen for their known roles in breast carcinogenesis (i.e., *PIK3CA* H1047R and E545K), potential as a therapeutic target in breast cancer (i.e., *PIK3CA* H1047R and E545K), potential to confer resistance to molecularly targeted therapies in breast cancer (i.e., *PIK3CA* H1047R and E545K, and *KRAS* G12D and G12V), or rare frequencies reported in breast cancer (*HRAS* G12D and *BRAF* V600E). By analyzing hotspot CDMs that are reported to be both common and uncommon in breast cancers, this study explored the prevalence of lower mutant frequency tumor subpopulations of prevalent breast cancer mutations (e.g., *PIK3CA* mutations) and determined whether mutations rarely detected in breast cancers using low-sensitivity methods (e.g., *KRAS* mutations) are found in breast cancers using a high-sensitivity mutation detection method. Our ACB-PCR results confirmed the presence of multiple CDMs in both normal breast and IDCs, suggesting that preexisting hotspot CDMs in normal breast may drive tumor initiation through clonal cooperation with other transformed cells (genetically and/or epigenetically). Furthermore, acquisition of subsequent CDMs, along with clonal expansion/contraction and possible recruitment of other transformed cells, culminates in the extensive intratumoral genetic heterogeneity observed in IDCs. Most CDMs quantified were at levels below the sensitivity of standard mutation detection methodologies, suggesting the potential for undetected mutant subpopulations to impact therapeutic response in the breast may be grossly underestimated.

Here, we extended our analysis of low-frequency CDMs in breast cancer subtypes, including TNBC. Specifically, we quantified six hotspot CDMs (*PIK3CA* H1047R, *PIK3CA* E545K, *KRAS* G12D, *KRAS* G12V, *HRAS* G12D, and *BRAF* V600E) in 81 IDCs (approximately 20 samples for each of the four breast cancer subtypes), using the sensitive and quantitative ACB-PCR approach. Mutant fractions (MFs) of the six CDMs were quantified in IDCs, analyzed by breast cancer subtype, and compared to levels measured in normal breast tissue [7,11].

## 2. Results

### 2.1. ACB-PCR Data Collection

**Figure 1:**
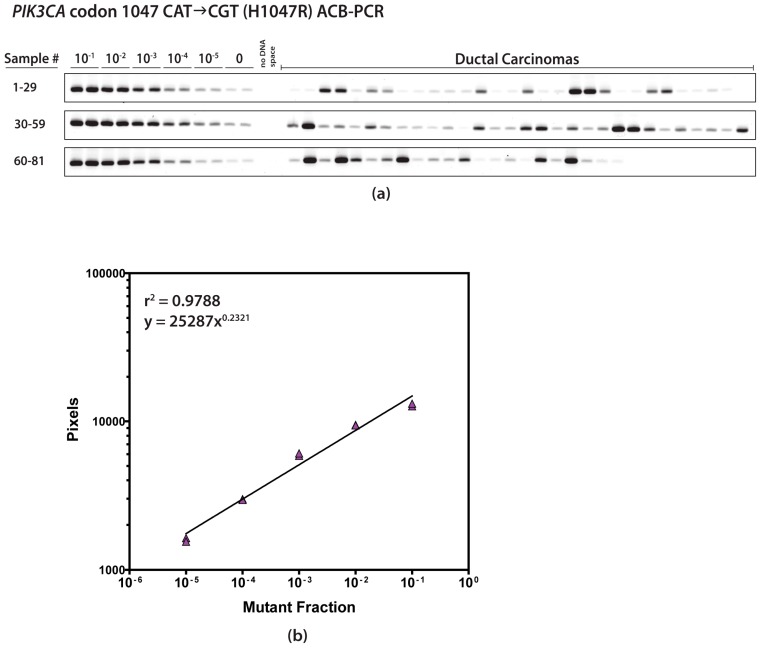
Representative images of the fluorescein-labeled ACB-PCR products on a polyacrylamide gel are shown for *PIK3CA* codon 1047 CAT→CGT (H1047R) (**a**). The pixel intensities of the bands produced from the standards were quantified and used to construct a standard curve relating pixel intensity to MF (**b**). The standard curve was used to interpolate the MFs of the samples from their measured fluorescence.

In total, 81 IDCs, including 20 HR+/HER2+, 20 HR+/HER2−, 21 HR−/HER2+, and 20 HR−/HER2− (TNBC) were characterized in terms of MF levels for six CDMs: *PIK3CA* H1047R and E545K, *KRAS* G12D and G12V, *HRAS* G12D, and *BRAF* V600E. Each sample was quantified in three independent experiments, except for samples 5, 10, 67, and 79, with only two *HRAS* G12D measurements. Thus, the ACB-PCR MF measurements for six targets in 81 IDCs generated a dataset of 1454 individual ACB-PCR measurements. The average coefficient of variation for the replicate *PIK3CA* H1047R, *PIK3CA* E545K, *KRAS* G12D, *KRAS* G12V, *HRAS* G12D, and *BRAF* V600E MF measurements were 0.81, 0.43, 0.61, 0.77, 0.79, and 0.62. An example of the ACB-PCR output, including the polyacrylamide gel and standard curve, is given in Figure 1.

### 2.2. Clinicopathological Characteristics of IDC Tissue Donors

The donors of IDC samples include 72 Caucasians (88.8%), five African Americans (6.2%), and four donors of unknown race (4.9%). Information regarding donor’s age, maximum tumor size, and histological grade were provided in pathology reports supplied by the repository for 80/81 samples. Tumor stage (T), and nodal stage (N) were provided for 77/81 samples. The mean age and maximum tumor dimension were 56.6 ± 12.5 years and 2.9 ± 2.1 cm, respectively. Clinical stage was discernable for 79/81 of the samples, which consisted of 18/81 (22.2%) stage I, 41/81 (50.6%) stage II, and 20/81 (24.7%) stage III samples. Information regarding smoking history and menopausal status was provided in chart reviews for 51/81 and 67/81 samples, respectively. Information regarding neoadjuvant and radiation therapy or past exposures was largely unavailable. A summary of donor and tumor characteristics (based on available data) for each subtype is given in Table 1.

### 2.3. MF Measurements in IDCs and IDC Subtypes

**Figure 2:**
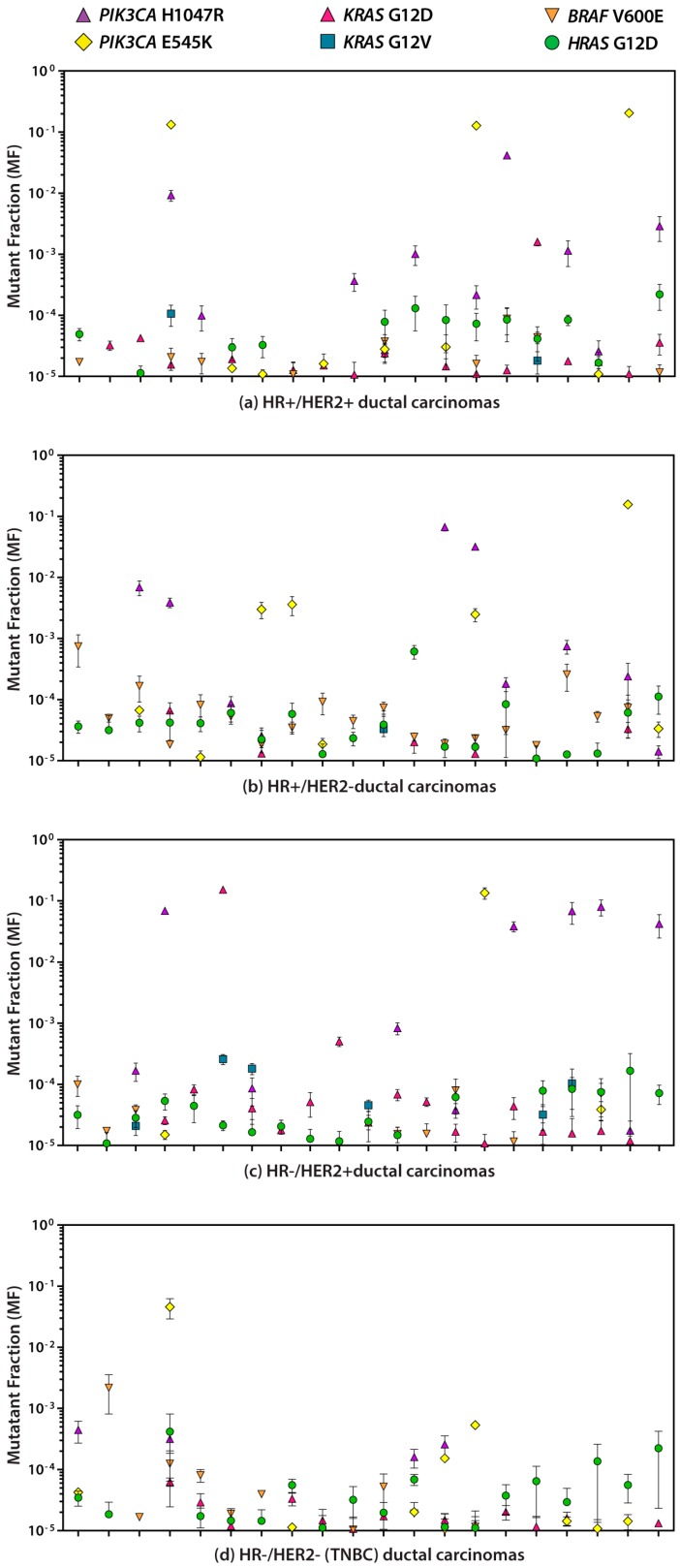
Levels of somatic mutations were quantified in (**a**) HR+/HER2+, (**b**) HR+/HER2−, (**c**) HR−/HER2+, and (**d**) HR−/HER2− (TNBC) IDCs. Error bars indicate the SEM for the replicate ACB-PCR MF measurements of each sample. Measurements below the level of accurate ACB-PCR quantification (1 × 10^−5^) are not shown.

The *PIK3CA* H1047R and E545K, *KRAS* G12D and G12V, *HRAS* G12D, and *BRAF* V600E mean MF measurements for each IDC are plotted in Figure 2A for HR+/HER2+ (*n* = 20), Figure 2B for HR+/HER2− (*n* = 20), Figure 2C for HR−/HER2+ (*n* = 21), and Figure 2D for HR−/HER2− (TNBC; *n* = 20). Measurable levels of mutation (i.e., ≥ 1 × 10^−5^, the lowest ACB-PCR standard employed) of the *PIK3CA* H1047R, *PIK3CA* E545K, *KRAS* G12D, *KRAS* G12V, *HRAS* G12D, and *BRAF* V600E were observed in 34/81 (42%), 29/81 (36%), 51/81 (63%), 9/81 (11%), 70/81 (86%), and 48/81 (59%) of IDCs, respectively. The geometric mean MFs for each of the six targets in IDCs and four IDC subtypes are given in Table 2. A summary of the ACB-PCR quantification of each target mutation for the individual samples within the four subtypes is given in Table S1A–D.

#### 2.3.1. Cancer-Driver Gene MFs in IDCs

**Figure 3:**
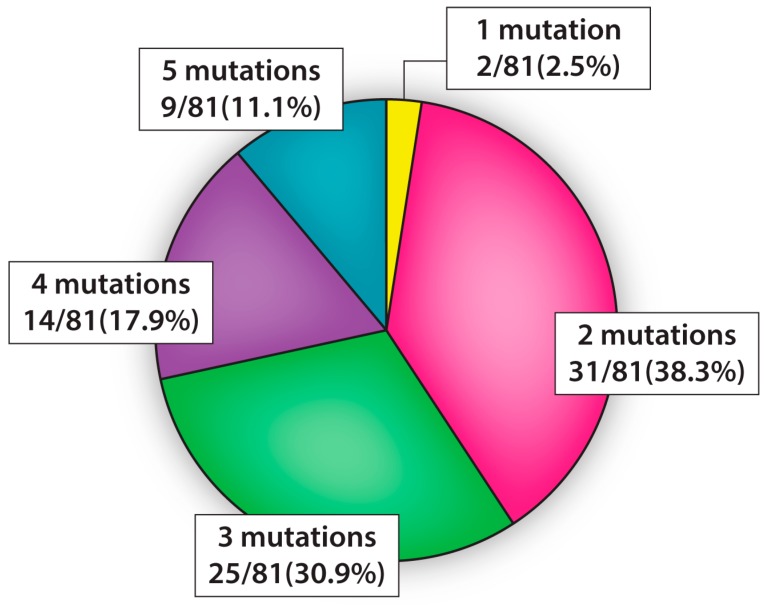
Percent of IDC samples with one, two, three, four, or five CDMs present at levels ≥ 1 × 10^−5^.

All IDCs encompassed at least one cancer-driving point mutation and most harbored multiple CDMs (at levels ≥ 1 × 10^−5^; Figure 3). Two CDMs were found in 31/81 (38.3%), three mutations in 25/81 (30.9%), four mutations in 14/81 (17%), and five mutations in 9/81 (11%) of IDCs. No IDC possessed all six CDMs at detectable levels. Surprisingly, this results in 98% of IDCs having ≥ 2, 59% having ≥ 3, and 28% having four or more of the measured CDMs present at levels ≥ 1 × 10^−5^. A description of the co-occurrence of hotspot point mutations in IDCs is given in Table 3. For example, Table 3 shows nearly 30% of IDCs carry both *KRAS* G12D and *BRAF* V600E mutations.

#### 2.3.2. Cancer-Driver Gene MFs in IDC Subtypes

**Figure 4:**
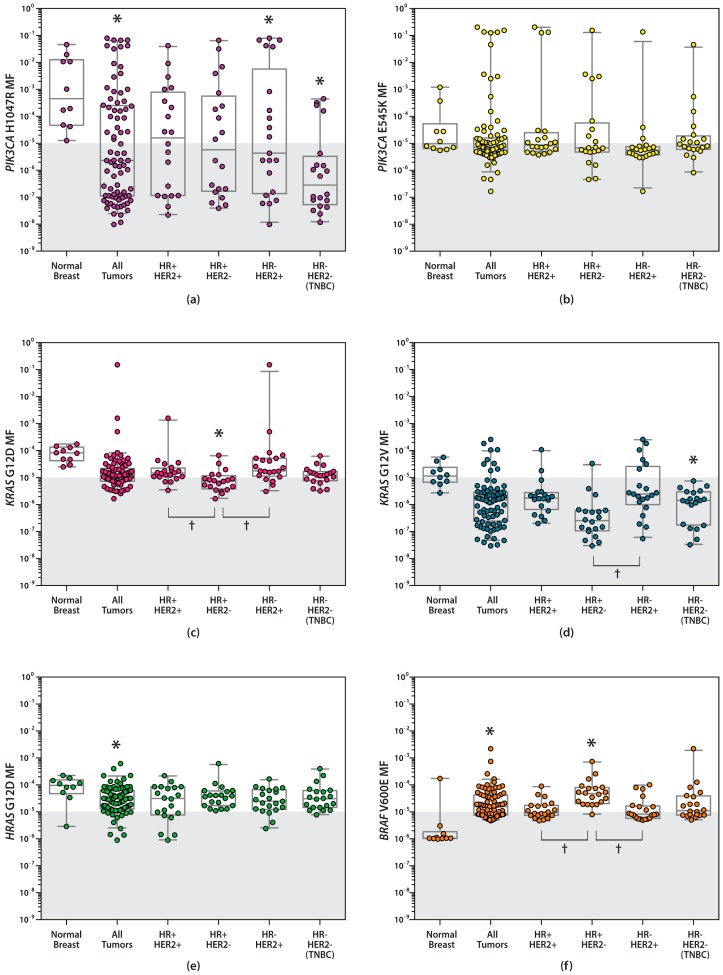
Dot plots and corresponding boxplots of (**a**) *PIK3CA* H1047R, (**b**) *PIK3CA* E545K, (**c**) *KRAS* G12D, (**d**) *KRAS* G12V, (**e**) *HRAS* G12D and (**f**) *BRAF* V600E MFs measured in normal breast [7,11] and four subtypes of IDC. Measurements in the shaded area are below the level of accurate ACB-PCR quantification (1 × 10^−5^). * Significant compared to normal breast. ^†^ Significant difference between IDC subtypes (Fisher’s exact test; *p* > 0.05).

A comparison of the distribution of measurements for *PIK3CA* H1047R, *PIK3CA* E545K, *KRAS* G12D, *KRAS* G12V, *HRAS* G12D, and *BRAF* V600E MFs in all IDCs for the four subtypes is given in Figure 4, along with MF measurements previously reported in normal breast (collected from individuals who died from causes unrelated to cancer or diseases affecting the breast [7,11]). Significant differences in the proportion of MFs above the level of accurate quantification were observed between normal breast and IDCs for *PIK3CA* H1047R, *BRAF* V600E, and *HRAS* G12D. Only the *BRAF* V600E mutation showed a significant increase compared to normal breast (Fisher’s exact test, *p* = 0.0049). For *PIK3CA* H1047R, and *HRAS* G12D, the proportion of MFs above the level of accurate quantitation were significantly lower when compared to normal breast (*p* = 0.0005, and 0.0235, respectively).

Few significant differences in *PIK3CA*, *KRAS*, *HRAS*, and *BRAF* MFs were observed between normal breast and IDC subtypes. Fisher’s exact test was used to test for difference in the number of MFs above the limit of detection (LOD) between pairwise groups of interest. While the geometric mean MF of *PIK3CA* H1047R for every subtype was more than 10-fold lower than that measured in normal breast (8.03 × 10^−4^ [7]), only the HR−/HER2+ and HR−/HER2− (TNBC) subtypes were significantly different (*p* = 0.0441 and *p* = 0.0003, respectively). The proportions of *KRAS* G12D MFs above the LOD were significantly different between HR+/HER2− and normal breast (*p* = 0.002). There were also significant differences between the HR+/HER2− subtype and the HR+/HER2+ and HR−/HER2+ subtypes (*p* = 0.0099 and 0.0046, respectively). For *KRAS* G12V, the geometric mean MF in the HR−/HER2− and HR+/HER2− subtypes were 14- to 40-fold less than that found in normal breast (1.23 × 10^−5^ [7]). However, significance was only reached in the HR−/HER2− subtype (*p* = 0.0177). *BRAF* V600E MFs were quantified and found to be greater in all four subtypes of IDCs as compared to normal breast [7]. However, only the HR+/HER2− subtype reached statistical significance (*p* = 0.0001) when compared to normal breast. In addition, *BRAF* V600E demonstrated significant difference when comparing HR+/HER2− to the HR+/HER2+ and HR−/HER2+ subtypes (*p* = 0.01 and 0.0015, respectively). No significant differences in *PIK3CA* E545K and *HRAS* G12D MFs were observed between normal breast ([7] and [9], respectively) and the IDC subtypes, or among each subtype.

#### 2.3.3. Cancer-Driver Gene MFs in IDCs Based on HR and HER2 Status

A comparison between cancer-driver MFs in IDCs that do or do not express HR and HER2 showed few significant differences. In HER2+ IDCs, the proportion of *KRAS* G12D and G12V MFs above the LOD were significantly greater compared to HER− tumors (*p* = 0.0012 and 0.0291, respectively). In contrast, in HER2− IDCs, a greater proportion of *BRAF* V600E MFs above the LOD were found compared to HER2+ IDCs (*p* = 0.0014). A moderate negative association in *KRAS* G12D and G12V, and *BRAF* V600E mutation was found in HR+ versus HR− IDCs, with a greater proportion of *KRAS* G12D MFs above the LOD in HR− IDCs, while *BRAF* V600E MFs above the LOD were observed in greater proportions in IDCs that are HR+, though the results were not significant.

### 2.4. Correlation Analyses

Exploratory correlation analyses using Spearman’s correlation coefficient were performed between CDMs, and between CDMs and clinicopathological features in IDCs and IDC subtypes. Additional studies are needed to confirm the association between various relationships.

#### 2.4.1. Correlation Analyses of CDMs in IDCs and IDC Subtypes

Correlation analyses revealed a few significant monotonic relationships between specific cancer-driver MFs in breast IDCs (weak to moderate in strength). Correlation analyses excluded MF measurements below our limit of accurate quantification (MFs < 1 × 10^−5^), thus limiting the number of samples analyzed for some CDMs (i.e., *KRAS* G12V), and, in some cases, resulted in insufficient numbers for analysis. The results are presented in a correlation matrix (Figure S1A–E; includes *p* and *r* values for each). For all IDCs (Figure S1A; *n* = 81), no significant correlation was observed between any two CDMs measured in this study. Correlation analysis within the individual subtypes revealed a positive association between *PIK3CA* H1047R and *HRAS* G12D and *PIK3CA* E545K mutations in the HR+/HER2+ subtype (Figure S1B). In the HR−/HER2+ subtype, *HRAS* G12D mutation was inversely associated with *KRAS* G12D mutation (Figure S1D). In the HR+/HER2− and HR−/HER2− (TNBC) subtypes, no significant correlations were observed between any CDMs; however, the correlation between *KRAS* G12D and *BRAF* V600E mutations in the HR−/HER2− subtype may warrant further investigation (*r* = 0.75, *p* = 0.0663; Figure S1E).

#### 2.4.2. Correlation Analyses of CDMs in IDCs Based on HR and HER2 Status

Correlation analyses of *PIK3CA*, *KRAS*, *HRAS*, and *BRAF* CDMs in IDCs that do or do not express HR and HER2 showed only a few weak, but significant, monotonic relationships (Figure S1F–I). No significant associations were observed in HR+ IDCs, however, in HR− IDCs, a positive association was observed between *BRAF* V600E and *HRAS* G12D MFs (Figure S1G). In HER2+ IDCs, negative associations included *KRAS* G12D and *HRAS* G12D MFs, as well as *KRAS* G12D and *PIK3CA* E545K MFs (Figure S1H). In the HER2− IDCs, a positive association between *BRAF* V600E and *KRAS* G12V MFs were found (Figure S1I).

#### 2.4.3. Correlation Analyses of CDMs and Clinicopathological Features of IDCs and IDC Subtypes

**Figure 5:**
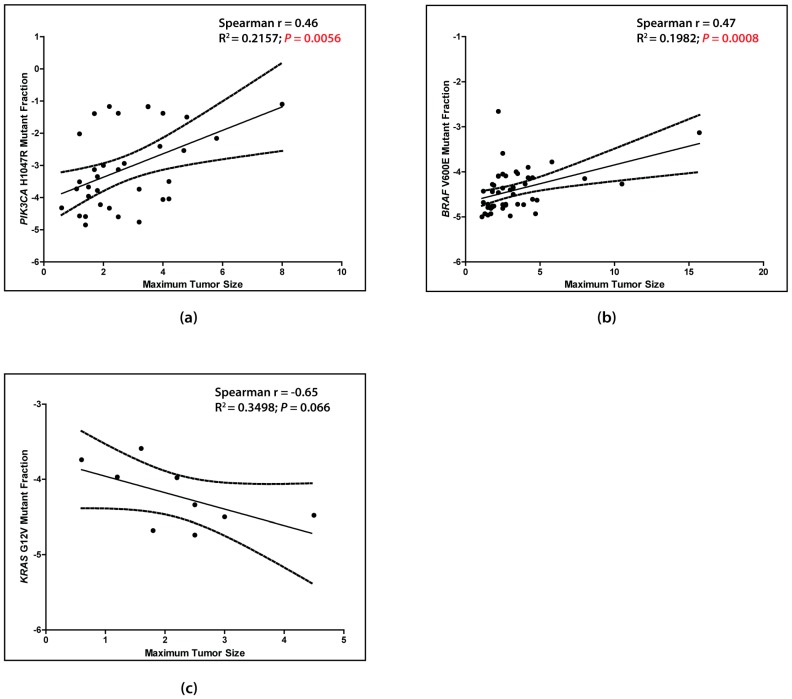
*PIK3CA* H1047R (**a**) and *BRAF* V600E (**b**) MFs are correlated positively with maximum tumor dimension. *KRAS* G12V (**c**) MF is correlated negatively with maximum tumor dimension (non-significant). Correlation analyses were conducted using samples with MFs ≥ 1 × 10^−5^. The solid line denotes the linear regression line. Dashed lines denote the 95% confidence interval. *p* values ≤ 0.05 are in red.

Using the available clinicopathological information for all IDCs in this study, correlation analyses between age, clinical stage, nodal involvement, menopausal status, maximum dimension, and histological grade, and the *PIK3CA*, *KRAS*, *HRAS*, and *BRAF* CDM levels measured by ACB-PCR were performed (Table 4). In all IDCs, for example, correlation analyses found *PIK3CA* H1047R MF was correlated positively with donor age and menopausal status (pre-menopausal versus post-menopausal (*r* = 0.48, *p* ≤ 0.0001; and *r* = 0.74, *p* = 0.0001, respectively)). *PIK3CA* H1047R and *BRAF* V600E MFs were correlated positively with maximum tumor dimension (*r* = 0.46, *p* ≤ 0.01; and *r* = 0.47, *p* ≤ 0.001, respectively), while *KRAS* G12V MF was correlated negatively with maximum tumor dimension (*r* = −0.65), although this did not reach the level of statistical significance (Table 4, Figure 5; using only samples with a MF ≥ 1 × 10^−5^).

In IDC subtypes, few significant associations between the clinicopathological characteristics and the CDMs measured were observed (Table 4). For example, in HR+/HER2+ IDCs, a significant positive correlation was found between *PIK3CA* H1047R mutation level and the donor’s clinical stage and menopausal status (*r* = 0.84, *p* ≤ 0.001; and *r* = 0.80, *p* ≤ 0.001, respectively). In HR+/HER2− tumors, *HRAS* G12D MF positively correlated with clinical stage and nodal involvement (*r* = 0.66, *p* ≤ 0.001; and *r* = 0.75, *p* ≤ 0.001, respectively). In HR−/HER2+ tumors, *KRAS* G12D MF was positively correlated with menopausal status (*r* = 0.81, *p* ≤ 0.001), while a negative correlation was observed between *KRAS* G12D MF and clinical stage (*r* = −0.81, *p* ≤ 0.001). *PIK3CA* E545K and *KRAS* G12D MFs showed positive and negative correlations with clinical stage (*r* = 0.98, *p* ≤ 0.001; and *r* = −0.83, *p* ≤ 0.001, respectively). *BRAF* V600E MF was positively correlated with clinical stage and overall histological grade in HR−/HER2+ tumors (*r* = 0.72, *p* ≤ 0.001; and *r* = 0.94, *p* ≤ 0.001, respectively). In HR−/HER2− (TNBC) tumors, *PIK3CA* E545K MF was positively correlated with nodal involvement (*r* = 0.96, *p* ≤ 0.001), and *HRAS* G12D MF was positively correlated with menopausal status and maximum tumor dimension (*r* = 0.85, *p* ≤ 0.001; and *r* = 0.55, *p* ≤ 0.05, respectively).

## 3. Discussion

The identification of oncogenic mutations and the use of that information in the care and management of cancer patients has become an important area of clinical investigation, driven by the precision medicine paradigm. While the genetics of familial breast cancer is well studied, remarkably few quantitative analyses have been done with respect to somatic mutations involved in sporadic breast cancer. Women diagnosed with TNBC face a poor prognosis and less effective treatment options than women with other breast cancer subtypes [19,20,21]. Improved understanding of the molecular genetic underpinnings of TNBC, as compared with other subtypes of breast cancer, may lead to better predictions of tumor behavior and treatment response. To elucidate the roles of low-frequency CDMs in breast cancer, a sensitive and quantitative approach called ACB-PCR was employed to characterize the frequency and distribution of six CDMs between the four subtypes of breast cancer, including HR+/HER2+, HR+/HER2−, HR−/HER2+, and HR−/HER2− (TNBC). The six CDMs included *PIK3CA* H1047R and E545K, *KRAS* G12D and G12V, *HRAS* G12D, and *BRAF* V600E. These mutations were chosen because of their known roles in breast carcinogenesis (e.g., mutations in *PIK3CA*), likelihood to impact targeted therapies currently being explored in breast cancer (e.g., mutant *PIK3CA* impacts resistance to HER2 targeted therapies; and mutations in key genes of the MAP kinase pathway that impact resistance to EGFR-targeted therapies (e.g., *KRAS*, *HRAS*, and *BRAF*)), or their potential as a therapeutic target (e.g., *PIK3CA* and *BRAF* mutant proteins).

This study has resulted in several critical findings, including the determination that: (1) all IDCs contain measurable levels of at least one CDM, with the majority (59%) harboring three or more of the six CDMs, (2) CDMs are present in a subpopulation of cells, and therefore, would go undetected by standard DNA sequencing, (3) *PIK3CA* H1047R and *BRAF* V600E are correlated positively with maximum tumor dimension, whereas *KRAS* G12V is correlated negatively (non-significant), and (4) subtype specific differences in MFs exist, most notably lower *PIK3CA* H1047R MFs in TNBCs (as compared to normal breast [7]).

Activating somatic mutations in *PIK3CA* are considered early, initiating events in breast cancer, resulting in cell proliferation and resistance to apoptosis. Furthermore, transgenic mouse models were used to demonstrate that expression of mutant *PIK3CA* induces mammary epithelial cell transformation and mouse mammary tumors [22]. *PIK3CA* is the most frequently mutated gene in human breast cancer, detected in 27% of breast carcinomas in the COSMIC database [6]. Three major hotspot mutations in *PIK3CA* are reported in IDC tumor samples, the H1047R, E545K, and E542K mutations, with reported mutation frequencies of 10.52%, 5.41%, and 3.22%, respectively. Data obtained using the sensitive and quantitative ACB-PCR approach generated a number of interesting findings and expanded current knowledge regarding *PIK3CA* mutation in human breast tumors. Specifically, (1) 59% of IDCs have measurable levels (≥ 1 × 10^−5^) of *PIK3CA* mutation, with 42%, 36%, and 19% having measurable levels of the *PIK3CA* H1047R, E545K, or both mutations, respectively, (2) *PIK3CA* H1047R and E545K exist as mutant subpopulations in IDCs, meaning they have levels of *PIK3CA* mutation that would go undetected by DNA sequencing, and (3) *PIK3CA* H1047R MF measurements in TNBC are significantly lower than that measured in normal breast. A greater level of *PIK3CA* mutation in normal breast than in breast tumors is consistent with the ideas that *PIK3CA* mutations are early drivers of multiclonal tumor origin, that multiple mutations are needed to overcome normal tissue homeostatic mechanisms, and that clonal selection and metapopulation dynamics drive the outgrowth of tumor cells, which may be predominantly other than *PIK3CA*-mutant (yet retaining minor *PIK3CA* mutant subpopulations), although some percentage of the time predominantly *PIK3CA*-mutant tumors do arise [23].

The high prevalence of mutated PIK3CA in breast cancer has created an active area of research as well as an attractive drug target. Mutation in the *PIK3CA* gene confers sensitivity to PI3K/AKT/mTOR pathway inhibition. Therefore, targeting components of this pathway has become a popular strategy in breast cancer therapeutics, particularly for use in TNBC [24,25,26]. Data generated from this study suggests that inhibitors of the PI3K/AKT/mTOR pathway may be a viable strategy for many breast cancer patients, considering 59% of IDCs had measurable levels of *PIK3CA* mutation, and 26% of IDCs had levels of *PIK3CA* mutation ≥ 1 × 10^−3^. For TNBC, however, very few samples had appreciable levels of *PIK3CA* mutation. Only four samples had a *PIK3CA* MF ≥ 1 × 10^−5^ and only one sample had a level of mutation ≥ 1 × 10^−3^, suggesting inhibition of the PI3K/AKT/mTOR pathway in TNBCs may not be a broadly effective strategy. Nevertheless, TNBC is not one homogeneous disease [27,28], suggesting the use of sensitive mutation detection approaches could be used to identify TNBCs with actionable *PIK3CA* mutations, such as those found in this study. Given the reported overexpression of EGFR in TNBC [29], and the relatively low levels of therapeutic resistance-causing *PIK3CA*, *KRAS*, and *BRAF* mutations found in TNBC, therapies directed against the EGFR receptor may be a viable avenue for further exploration.

Previously, we showed surprisingly high levels of the *PIK3CA* H1047R mutation were present in normal breast tissue (median MF = 6.13 × 10^−4^; range = 1.25 × 10^−5^ − 4.59 × 10^−2^) [7]. Interestingly, we found many tumors (~ 60%) in this study contained *PIK3CA* H1047R mutation at levels lower than the lowest *PIK3CA* H1047R MF measured in normal breast. We hypothesize that pre-existing *PIK3CA* mutant cells can drive tumor initiation and early progression but are lost or outgrown by other cancer driving clones in most advanced breast cancers. Data from the COSMIC database supports this idea, with *PIK3CA* mutation detected in 49% of hyperplasia, 29% of DCIS, and 24% in IDCs [6], suggesting the level of *PIK3CA* mutation decreases as disease progression ensues. In the future, it would be interesting to analyze spatially-disparate normal samples from individuals with IDC and compare levels of mutations between normal and tumor samples and between normal breast samples from women with and without IDC.

Although found at lower frequencies (as compared to mutations in *PIK3CA*), *KRAS*, *HRAS*, and *BRAF* mutations were highly prevalent across IDC subtypes, albeit as minor mutant subpopulations. Measurable levels of *KRAS* G12D and G12V, *HRAS* G12D and *BRAF* V600E were found in 63%, 11%, 84%, and 59% of IDCs, respectively. This is in stark contrast to the reported frequencies in the COSMIC database, where *KRAS* G12D and G12V, *HRAS* G12D, and *BRAF* V600E are reported to occur in 8/2585 (0.31%), 4/2585 (0.16%), 0/2159 (0%), and 0/1844 (0%) of IDC tumor samples [6]. The finding that minor mutant subpopulations exist in breast IDCs may have therapeutic implications, as mutations in the genes of the MAP kinase pathway are known to impact response to molecularly-targeted therapy for other cancer types [30,31,32,33,34]. Interestingly, an inverse relationship between *KRAS* and *BRAF* mutation immerged in this dataset. That is, (1) *BRAF* V600E and *KRAS* G12D as well as *BRAF* V600E and *KRAS* G12V MFs were inversely correlated in IDCs, (2) *BRAF* V600E was correlated positively with maximum tumor dimension, whereas *KRAS* G12V MF was correlated negatively (non-significant), (3) greater *BRAF* V600E MFs were observed in the HR+/HER2− subtype compared to the HR+/HER2+ and HR−/HER2+ subtypes, whereas significantly less *KRAS* G12D mutation was observed in the HR+/HER2− subtype as compared to HR+/HER2+ and HR−/HER2+ subtypes, and (4) *BRAF* V600E was significantly greater in HER2− samples, while *KRAS* (G12D and G12V) were significantly greater in HER2+ IDCs. The inverse nature of the prevalence of the *BRAF* and *KRAS* mutations may be due, in part, to redundancy in the MAP kinase pathway. While *BRAF* and *KRAS* mutations have been described as “mutually exclusive” [35], we observed 26/81 (32.1%) of IDCs contained mutations in both genes. It is currently unclear what impact these low-frequency mutant subpopulations of critical genes in the MAP kinase pathway may have on tumor biology and therapeutic response.

A growing body of evidence suggests that undetected mutant subpopulations may impact response to molecularly targeted cancer therapies [30,31,36,37]. For example, in HER2-overexpressing breast cancer, mutations in *PIK3CA* have been shown to confer resistance to the HER2-targeted therapy, trastuzumab [38,39]. In this study, measurable levels of *PIK3CA* mutation (H1047R, E545K or both) were found in 63.4% of HER2+ IDCs, as compared to 15.6% reported in the COSMIC database. The high prevalence of *PIK3CA* mutant subpopulations may explain, in part, the high frequency of relapse in these patients after trastuzumab therapy. Furthermore, we observed that most breast IDCs contain multiple CDMs. For example, 53.0% of IDCs contained clonal subpopulations expected to be PIK3CA and MAP kinase pathway mutant, suggesting that combinations of molecularly-targeted therapies may be required for adequate treatment of breast cancer.

The underappreciated role of MAP kinase pathway gene mutations in breast cancer pathology and response to treatment may be due, in part, to the use of insensitive mutation detection methodologies in an exceedingly heterogenous tumor. Most mutation detection techniques employed today (i.e., DNA sequencing, ARMS/Scorpions, MALDI-TOF, mutant-enriched PCR, and high-resolution melting analysis) do not have the required sensitivity to detect minor mutant subpopulations present in a heterogeneous tumor, such as breast cancer [37]. ACB-PCR is an exceptionally powerful and unique technique, shown to quantify particular cancer-related base pair substitution mutations in normal human tissues and tumors with a sensitivity of 1 × 10^−5^ [7,8,11,15,30,40,41]. Only mutation detection techniques with this level of sensitivity would be able to adequately characterize the gene mutations in the MAP kinase pathway quantified in this study.

One major limitation of this study is the lack of adequate samples representing African-American women. Breast cancers in African-American women display different characteristics than in Caucasian women [42]. In African-American women, breast cancers (1) occur with an earlier onset, (2) show less favorable outcomes, and (3) present with a more aggressive tumor phenotype [43]. Many differences have been attributed to these disparities, most notably the higher prevalence of TNBC in women of African-American decent [44,45,46,47]. While differences in epidemiology and prognosis between African-American and Caucasian women with breast cancer (and most notably TNBC) have been described in the literature [44,47], few data are available regarding possible differences in the somatic mutations that may underlie these differences. Therefore, ACB-PCR analyses to detect potential differences in low frequency somatic point mutations in TNBC of women of African origin are ongoing.

## 4. Materials and Methods

### 4.1. IDCs Sample Collection

Procedures for the acquisition and analysis of pre-existing, anonymous human tissues were reviewed by the FDA’s IRB (Research Involving Human Subjects Committee (RIHSC), FWA 00006196; RIHSC Protocol #12-010T, original approval date: 02/27/2012). Eighty-one fresh-frozen female primary breast IDCs (including 20 HR+/HER2+, 20 HR+/HER2−, 21 HR−/HER2+, and 20 HR−/HER2− (TNBC)) were purchased from the National Cancer Institute’s Cooperative Human Tissue Network or Asterand (Detroit, MI, USA). All IDCs were histologically evaluated by board certified pathologists, who confirmed the original diagnosis. All IDCs were stored at −80 °C until DNA extraction.

### 4.2. DNA Isolation

All IDCs were frozen in liquid nitrogen and pulverized using mortar and pestle. The pulverized samples were further homogenized using an Omni THQ tissue homogenizer (Omni International, Kennesaw, GA, USA) and 6 mL proteinase K buffer [1 mg/mL proteinase K, 100 mM NaCl, 25 mM EDTA (pH 8), and 1% SDS] per gram of tissue. The homogenate was incubated ~16 h at 37 °C, and DNA extraction completed as described previously [40].

### 4.3. First-Round PCR

First-round PCR products of *PIK3CA* codon 545, *KRAS* codon 12, *HRAS* codon 12, and *BRAF* codon 600 were generated using high-fidelity *PfuUltra* Hotstart DNA Polymerase (Agilent Technologies, Santa Clara, CA, USA) and 1 µg of EcoRI-digested genomic DNA as described previously [7,11]. For *PIK3CA* codon 1047, generation of the 308 base-pair product was performed using 1 µg of EcoRI-digested genomic DNA in a 200 µL PCR reaction containing: 1× *PfuUltra* HF Reaction Buffer [10 mM KCl, 10 mM (NH_4_)_2_SO_4_, 20 mM Tris-HCl (pH 8.75), 2 mM MgSO_4_, 0.1% Triton X-100, 0.1 mg/mL bovine serum albumin; Agilent Technologies], 0.2 mM dNTPs, 0.2 µM forward primer (5′-ACATCATTTGCTCCAAACTGAC-3′), 0.2 µM reverse primer (5′-ATGCATGCTGTTTAATTGTGT-3′), and 10 units of cloned *PfuUltra* Hotstart DNA Polymerase (Agilent Technologies). PCR reactions were carried out using DNA Engine or DNA Engine Tetrad thermocyclers (Bio-Rad, Hercules, CA, USA) with the following cycling conditions: 2 min denaturation at 94 °C, followed by 32 cycles of 1 min at 94 °C, 1 min at 54 °C, and 1 min at 72 °C, and a final 7 min extension at 72 °C. All first-round PCR products were purified by ion-pair reverse phase chromatography using a WAVE Nucleic Acid Fragment Analysis System (Transgenomics Inc., Omaha, NE, USA), evaporated to dryness, and resuspended in TE buffer (5 mM Tris, 0.5 mM EDTA, pH 7.5). PCR products were frozen and stored at −80 °C as multiple single-use aliquots. The DNA concentration of each sample was determined by repeated measurements of the single-use aliquots using an Epoch Spectrophotometer (BioTek, Winooski, VT, USA). Final DNA concentrations of each amplicon were calculated from three measurements that varied by ≤10% from the group mean.

### 4.4. Generation of Mutant and Wild-Type Standards and ACB-PCR

ACB-PCR is an allele-specific amplification method that can be used to selectively amplify and quantify a mutant allele in a 100,000-fold excess of wild-type allele. Each ACB-PCR assay essentially includes a reconstruction experiment. Mutant and wild-type plasmid DNAs are used as template to PCR amplify the same amplicon from the genomic sample DNAs in a high-fidelity first-round PCR, which is optimized to ensure equal number of duplications occur between the different sample types. Based on the DNA concentration of the mutant and wild-type amplicons, they are combined to generate a set of mutant fraction (MF) standards with the defined MFs of 10^−1^, 10^−2^, 10^−3^, 10^−4^, 10^−5^, and 0 (wild-type amplicon only). Equal numbers of molecules of the defined MF standards and unknowns are then analyzed by ACB-PCR. The signal generated from the wild-type-only amplicon defines the background signal of each ACB-PCR assay. The remaining MF standards are used to construct a standard curve, from which the MFs of the unknown samples can be interpolated [8]. Mutant *PIK3CA* H1047R (codon 1047 CGT) and E545K (codon 545 AAG), *KRAS* G12D (codon 12 GAT) and G12V (codon 12 GTT), *HRAS* G12D (codon 12 GAC), and *BRAF* V600E (codon 600 GAG) and wild-type (*PIK3CA* codon 1047 CAT and codon 545 GAG, *KRAS* codon 12 GGT, *HRAS* codon 12 GGC, and *BRAF* codon 600 GTG) standards were prepared by PCR amplification of cloned mutant or wild-type plasmid DNAs. For *PIK3CA* codon 545, *KRAS* codon 12, *HRAS* codon 12, and *BRAF* codon 600, primers and reaction conditions were identical to those used for amplification of the target sequences from genomic DNAs [7,11]. For *PIK3CA* codon 1047, extended upstream and downstream primers (up, 5′-ACATCATTTGCTCCAAACTGACCAAACTGTTCTTATTACTTA-3′; down, 5′-ATGCATGCTGTTTAATTGTGTGGAAGATCCAATCCATTTTTGT-3’) were used to generate a 308 bp product identical in size and sequence to the amplicon in the genomic DNAs. Purification, storage, and quantification of wild-type and mutant PCR products were performed as described above.

On the day of the ACB-PCR, purified *PIK3CA* codon 1047 and codon 545, *KRAS* codon 12, *HRAS* codon 12, or *BRAF* codon 12 mutant and wild-type reference DNAs (prepared by PCR amplification of cloned mutant or wild-type plasmid DNAs [7]) were mixed to generate standards with MFs of 10^−1^, 10^−2^, 10^−3^, 10^−4^, 10^−5^, and 0 (containing only the wild-type sequence), at a concentration of 5 × 10^7^ copies/μL for the *PIK3CA* E545K, *KRAS* G12D and G12V, and *BRAF* V600E standards, and 1 × 10^8^ copies/μL for the *PIK3CA* H1047R and *HRAS* G12D standards. Ten microliters of the standard DNA mixture or genomic DNA was used in the corresponding ACB-PCR reaction, for a total of 5 × 10^8^ copies per reaction for *PIK3CA* E545K, *KRAS* G12D and G12V, and *BRAF* V600E assays, and 1 × 10^9^ copies per reaction for *PIK3CA* H1047R and *HRAS* G12D assays. Duplicate reactions for each MF standard, along with a no-DNA control was included for every ACB-PCR experiment. ACB-PCR primers (including a fluorescein-labelled mutant-specific primer (MSP), a blocker-primer (BP), and an up- or downstream primer) and PCR reaction and cycling conditions were performed as previously described for *HRAS* G12D and *BRAF* V600E [7], and *PIK3CA* E545K [11].

For the *PIK3CA* H1047R ACB-PCR, each 50 μL reaction contained: 1× Standard Taq Buffer (New England Biolabs (NEB), Beverly, MA, USA), 1.5 mM MgCl_2_, 0.1 mg/mL gelatin, 1.0 mg/mL Triton X-100, 40 μM dNTPs, 150 nM MSP (5′-fluorescein-TGTTGTCCAGCCACCATGTC-3′), 500 nM BP (5′-TGTTGTCCAGCCACCATGTdT-3′), 150 nM upstream primer (5′-GATGCTTGGCTCTGGAATGC-3′), 60 mU PerfectMatch PCR Enhancer (Agilent Technologies), and 0.3 µL Hemo KlenTaq DNA polymerase (NEB). Cycling conditions were 2 min at 94 °C, followed by 41 cycles of 94 °C for 30 s, 49 °C for 45 s, and 72 °C for 1 min.

For measurement of *KRAS* G12D, each 50 μL reaction contained: 1× Standard Taq Buffer, 1.3 mM MgCl_2_, 0.1 mg/mL gelatin, 1.0 mg/mL Triton X-100, 80 μM dNTPs, 500 nM MSP (5′-fluorescein-CTTGTGGTAGTTGGAGCTTA-3′), 475 nM BP (5′-CTTGTGGTAGTTGGAGCTTdG-3′), 500 nM downstream primer (5′-GATTTACCTCTATTGTTGGA-3′), 80 mU PerfectMatch PCR Enhancer, and 0.5 µL Hemo KlenTaq DNA polymerase.

For the *KRAS* G12V ACB-PCR, each 50 μL reaction contained: 1× Standard Taq Buffer, 1.25 mM MgCl_2_, 0.1 mg/mL gelatin, 1.0 mg/mL Triton X-100, 40 μM dNTPs, 400 nM MSP (5′-fluorescein-CTTGTGGTAGTTGGAGCTAT-3′), 400 nM BP (5′-CTTGTGGTAGTTGGAGCTAdG-3′), 400 nM downstream primer (5′-GTTGGATCATATTCGTCCAC-3′), 90 mU PerfectMatch PCR Enhancer, and 0.4 µL Hemo KlenTaq DNA polymerase.

### 4.5. Vertical Polyacrylamide Gel Electrophoresis, Image Analysis, and Data Collection

Equal volumes of ACB-PCR products, containing 1× ficoll loading buffer/dye, were loaded on non-denaturing 8% vertical polyacrylamide gels. The fluorescent ACB-PCR products were visualized using a PharosFX Molecular Imager with an external blue laser (Bio-Rad). The pixel intensities of the ACB-PCR product bands (*PIK3CA* H1047R, 148 bp; *PIK3CA* E545K, 77 bp; *KRAS* G12D, 103 bp; *KRAS* G12V, 89 bp, *HRAS* G12D, 95 bp; and *BRAF* V600E, 98 bp) were quantified using Quantity One software (Bio-Rad) and a locally averaged background correction. For *PIK3CA* H1047R, *KRAS* G12D and G12V, *HRAS* G12D and *BRAF* V600E, construction of the standard curve and interpolation of the MF of unknowns was performed as previously described [7]. For *PIK3CA* E545K, the construction of a standard curve utilizing all MF standards (1 × 10^−5^ to 1 × 10^−1^) did not accurately interpolate the MF of the unknown samples at both the low and high end of the standard curve based on their respective fluorescent intensities, in pixels (i.e., overestimation at the low end and underestimation at the high end of MF). To circumvent an under- or overestimation, *PIK3CA* E545K MF was interpolated from standard curves that gave the best quantification across an observable range of MFs. Specifically, MF standards were used to construct two log-log plots; one 1 × 10^−5^ to 1 × 10^−3^ for samples with pixel intensities < 1 × 10^−3^ MF standard, and one 1 × 10^−3^ to 1 × 10^−1^ for samples with pixel intensities > 1 × 10^−3^ MF standard. MFs were calculated using the power function and pixel intensities of the *PIK3CA* E545K ACB-PCR products.

### 4.6. MF Measurements in Normal Breast

Ten fresh-frozen normal breast samples (collected at autopsy from individuals who died from causes other than cancer or breast disease) were purchased from the National Disease Research Interchange (NDRI; Philadelphia, PA, USA) [7,11]. DNA isolation, first-round PCR amplification of *PIK3CA* codon 1047 and 545, *KRAS* codon 12, *HRAS* codon 12 and *BRAF* codon 600, and subsequent ACB-PCR quantification of *PIK3CA* H1047R and E545K, *KRAS* G12D and G12V, *HRAS* G12D, and *BRAF* V600E MFs were described [7,11]. The MF measurements previously reported in normal breast have been integrated into the analyses of the current study.

### 4.7. Statistical Analyses

The arithmetic average of three independent MF measurements was used to calculate the MF of each target of interest for all samples. The average MF measurement for each sample was log_10_-transformed. For each mutational target, the geometric mean MF was calculated as the average log_10_-transformed MFs measured in each group. Log_10_-transformed data were used for all subsequent analyses. For group comparisons, non-parametric statistical methods were employed because the log-transformed data were not normally distributed. For comparisons between normal breast and all IDCs, Fisher’s exact test was employed to compare the numbers of samples above and below the levels of accurate ACB-PCR quantitation (i.e., > 1 × 10^−5^ and < 1 × 10^−5^), using GraphPad Prism 5 Software (GraphPad Software, Inc., La Jolla, CA, USA). Differences in cancer-driver MFs between IDC subtypes and normal breast tissue were assessed using Fisher’s exact test with Holm’s correction for multiple comparison testing. Correlation analyses were assessed using Spearman’s rank correlation coefficient (unadjusted for tied data). Correlation between a quantitative variable and an ordinal variable (e.g., MF and clinical stage) was assessed with polyserial correlation. For all statistical analyses, significance was defined as *p* ≤ 0.05.

## 5. Conclusions

The sensitive and quantitative ACB-PCR approach was used to obtain a detailed and quantitative molecular genetic analysis of six CDMs in 81 IDCs, categorized into four IDC subtypes (including TNBC). Significant differences in levels of these CDMs were observed among the four IDC subtypes, as well as that of normal breast tissue previously published. Of significant note, the *PIK3CA* H1047R mutation was found to be lower in TNBCs, suggesting a lack of benefit with PI3K/AKT/mTOR inhibition in these individuals. In HER2+ breast tumors, *PIK3CA* mutation (H1047R, E545K, or both) was found in 63% of tumors, which may subvert the efficacy of HER2-targeted therapies and ultimately result in relapse. Furthermore, a large percentage of breast cancers were found to have measurable levels of mutations in critical genes of the MAP kinase pathway (i.e., *KRAS*, *HRAS*, and *BRAF*). While the MAP kinase pathway gene mutations analyzed in this study have only rarely been associated with breast cancer, their roles may be underestimated, due to the use of relatively insensitive mutation detection methods. Thus, the low-frequency mutational profile of IDC subtypes, including TNBC, revealed in this study identified points in tumor-related pathways that are the most appropriate/inappropriate for therapeutic intervention. Furthermore, the data generated provide a rationale for incorporating more sensitive and quantitative approaches (in place of standard DNA sequencing) into future clinical trials designed to address specific personalized medicine questions.

## Figures and Tables

**Table 1:** Tissue donor and tumor characteristics according to subtype.

Characteristics	HR+/HER2+ (*n* = 20)	HR+/HER2− (*n* = 20)	HR−/HER2+ (*n* = 21)	HR−/HER2− (TNBC) (*n* = 20)	*P*
Age (mean ± SD)	55.75 ± 13.32	56.45 ± 14.24	58.24 ± 11.48	55.90 ± 11.63	0.9183 ^1^
Max. tumor dimension (mean ± SD)	2.26 ± 1.18	4.36 ± 3.29	2.51 ± 1.52	2.47 ± 0.96	0.0019 ^1^
Histological grade ^2^					0.0002 ^3^
I	0	4	0	1	
II	15	8	6	3	
III	5	7	15	15	
Tumor stage ^2^					0.0009 ^3^
T1	12	1	9	8	
T2	6	15	11	11	
T3	0	4	0	0	
T4	2	0	1	0	
Nodal stage ^2^					0.1181 ^3^
N0	12	8	10	12	
N1	4	4	6	4	
N2	3	4	4	2	
N3	0	4	0	0	
Clinical stage ^2^					0.0316 ^3^
I	7	0	7	4	
II	8	11	9	13	
III	4	9	5	2	
Ever smoked ^2^					0.0588 ^3^
Yes	1	7	4	7	
No	8	12	9	3	
Menopausal status ^2^					0.5140 ^3^
Pre-menopause	7	4	4	4	
Post-menopause	10	11	17	10	

^1^ ANOVA; ^2^ Data not available for all samples; ^3^ Chi-square test using available data.

**Table 2:** Geometric mean MFs ^1^ of six CDMs in IDCs and by subtype.

Mutation	HR+/HER2+ (*n* = 20)	HR+/HER2− (*n* = 20)	HR−/HER2+ (*n* = 21)	HR−/HER2− (TNBC) (*n* = 20)	All IDCs
*PIK3CA* H1047R	**1.32 × 10^−5^**	**1.20 × 10^−5^**	**2.15 × 10^−5^**	7.64 × 10^−7^	7.23 × 10^−6^
*PIK3CA* E545K	**3.66 × 10^−5^**	**2.29 × 10^−5^**	7.94 × 10^−6^	**1.68 × 10^−5^**	**1.81 × 10^−5^**
*KRAS* G12D	**1.79 × 10^−5^**	7.43 × 10^−6^	**3.47 × 10^−5^**	**1.18 × 10^−5^**	**1.55 × 10^−5^**
*KRAS* G12V	1.80 × 10^−6^	3.05 × 10^−7^	3.57 × 10^−6^	8.57 × 10^−7^	1.15 × 10^−6^
*HRAS* G12D	**2.09 × 10^−5^**	**3.59 × 10^−5^**	**2.54 × 10^−5^**	**3.34 × 10^−5^**	**2.82 × 10^−5^**
*BRAF* V600E	**1.19 × 10^−5^**	**4.84 × 10^−5^**	**1.19 × 10^−5^**	**1.96 × 10^−5^**	**1.90 × 10^−5^**

^1^ Geometric mean MFs were calculated using all MF measurements extrapolated from pixel intensities and standard curves, including measurements below the limit of accurate ACB-PCR quantification (1 × 10^−5^). Subtype geometric mean MFs greater than the limit of accurate ACB-PCR quantification are shown in bold. IDCs: invasive ductal carcinomas.

**Table 3:** Co-occurrence of hotspot CDMs in IDCs of the breast.

CDM	*PIK3CA* H1047R	*PIK3CA* E545K	*KRAS* G12D	*KRAS* G12V	*HRAS* G12D	*BRAF* V600E
*PIK3CA* H1047R	-	18.52%	27.16%	4.94%	37.04%	29.63%
*PIK3CA* E545K		-	23.46%	1.24%	30.86%	19.75%
*KRAS* G12D			-	8.64%	51.85%	29.62%
*KRAS* G12V				-	9.88%	4.94%
*HRAS* G12D					-	51.85%
*BRAF* V600E						-

**Table 4:** Spearman *r* correlation coefficient ^1^ between cancer-driver MFs ^2^ and clinicopathological characteristics of IDCs and IDC subtypes.

Group	CDM	Age	Clinical Stage	Nodal Involvement	Menopause Status	Maximum Tumor Dimension	Overall Hist. Grade
All DCs	*PIK3CA* H1047R	0.48 ***	0.28	−0.26	0.74 ***	0.46 **	−0.23
	*PIK3CA* E545K	0.14	0.12	0.31	−0.35	0.19	−0.17
	*KRAS* G12D	0.11	−0.33	0.09	0.72 ***	0.00	0.21
	*KRAS* G12V	−0.33	−0.41	−1.00 ***	NA	−0.65	−0.21
	*HRAS* G12D	0.09	0.32 **	0.27	−0.07	0.23	−0.19
	*BRAF* V600E	0.21	0.25	−0.17	0.15	0.47 ***	0.08
HR+/HER2+	*PIK3CA* H1047R	0.50	0.84 ***	0.44	0.80 ***	0.52	−0.45
	*PIK3CA* E545K	0.53	0.42	NA	0.30	0.35	0.29
	*KRAS* G12D	−0.02	0.18	0.58 *	0.33	0.30	0.44
	*KRAS* G12V	−1.00	NA	NA	NA	−1.00	−1.00
	*HRAS* G12D	0.23	0.18	0.08	0.07	0.33	0.15
	*BRAF* V600E	0.54	−0.31	−0.54	NA	0.06	0.36
HR+/HER2-	*PIK3CA* H1047R	0.01	−0.47	−0.58 *	0.93 ***	0.54	−0.38
	*PIK3CA* E545K	−0.31	−0.68 *	−0.07	−1.00 ***	0.05	−0.49
	*KRAS* G12D	−0.15	−0.27	0.27	−1.00	−0.40	0.13
	*KRAS* G12V	NA	NA	NA	NA	NA	NA
	*HRAS* G12D	−0.32	0.66 ***	0.75 ***	−0.54 *	0.01	−0.25
	*BRAF* V600E	0.36	0.40	0.03	0.58 *	0.28	0.09
HR-/HER2+	*PIK3CA* H1047R	0.51	0.50	−0.34	1.00	0.49	−0.39
	*PIK3CA* E545K	−0.50	0.98 ***	1.00	−1.00	−0.50	NA
	*KRAS* G12D	0.00	−0.83 ***	−0.56 *	0.81 ***	−0.53 *	0.26
	*KRAS* G12V	−0.94 *	−0.39	−1.00 ***	NA	−0.60	−0.33
	*HRAS* G12D	0.37	0.66	0.45	−0.10	0.62 **	0.08
	*BRAF* V600E	−0.19	0.72 ***	0.26	−0.27	0.68	0.94 ***
HR-/HER2- (TNBC)	*PIK3CA* H1047R	0.00	−0.12	NA	0.40	0.60	−0.56
	*PIK3CA* E545K	0.39	−0.01	0.96 ***	0.06	−0.14	−0.23
	*KRAS* G12D	−0.02	−0.19	0.05	0.46	0.04	−0.45
	*KRAS* G12V	NA	NA	NA	NA	NA	NA
	*HRAS* G12D	0.36	0.26	−0.05	0.85 ***	0.55 *	−0.23
	*BRAF* V600E	0.02	−0.03	−0.64 *	−0.42	0.48	0.04

^1^ Unadjusted for tied data; ^2^ Using only samples with a MF ≥ 1 × 10^−5^; * *p* ≤ 0.05; ** *p* ≤ 0.01; *** *p* ≤ 0.001. Hist. Grade: histological grade; NA: not analyzed due to insufficient number of samples with MFs ≥ 1 × 10^−5^.

## Supplementary Materials

Supplementary materials can be found at https://www.mdpi.com/1422-0067/20/5/1011/s1.

## Abbreviations

ACB-PCR	Allele-specific competitive blocker polymerase chain reaction
CDMs	CDMs
ER	Estrogen receptor
HR	Hormone receptor
HER2	Human epidermal growth factor receptor-2
MF	Mutant fraction
PR	Progesterone receptor
TNBC	Triple-negative breast cancer
