# Supplementary material for: Low-Frequency Mutational Heterogeneity of Invasive Ductal Carcinoma Subtypes: Information to Direct Precision Oncology
Source: Int J Mol Sci. 2019 Feb 26;20(5):1011. doi: 10.3390/ijms20051011 (PMC6429455; doi:10.3390/ijms20051011)
Supplement: Supplementary file 1 [file ijms-20-01011-s001.pdf]

International Journal of *Molecular Sciences*

MDPI

Supplementary Materials: Low-frequency mutational heterogeneity of invasive ductal carcinoma subtypes: information to direct precision oncology

Meagan B. Myers *, Karen L. McKim, Malathi Banda, Nysia I. George and Barbara L. Parsons

Table S1A *PIK3CA, KRAS, HRAS,* and *BRAF* mutant fraction measurements in HR+/HER2+ ductal carcinomas

						Mutant Fraction					
		Max. Tumor	T Stage ∣ N Stage ∣			*PIK3CA*	*PIK3CA*	*KRAS*	*KRAS*	*HRAS*	*BRAF*
ID	Age/Race	Dim.	Hist. Grade ∣ Clinical Stage	Smoker	MP	H1047R	E545K	G12D	G12V	G12D	V600E
1	40/C	1.8	T1 ∣ N1 ∣ II ∣ II	N	Pre	1.99×10^{-6}	6.99×10^{-6}	3.37×10^{-6}	5.88×10^{-7}	4.94×10^{-5}	1.72×10^{-5}
2	85/C	3.2	T4 ∣ N0 ∣ III ∣ III	-	Post	1.16×10^{-7}	4.37×10^{-6}	3.22×10^{-5}	2.29×10^{-6}	7.26×10^{-6}	5.71×10^{-6}
3	56/C	4.2	T4 ∣ N2 ∣ II ∣ III	-	Post	1.11×10^{-7}	4.06×10^{-6}	4.23×10^{-5}	2.57×10^{-7}	1.14×10^{-5}	8.62×10^{-6}
4	69/C	1.2	T1 ∣ N- ∣ II ∣ -	N	Post	9.21×10^{-3}	1.32×10^{-1}	1.56×10^{-5}	1.06×10^{-4}	1.44×10^{-6}	2.07×10^{-5}
5	43/C	1.9	T1 ∣ N0 ∣ II ∣ I	-	Pre	9.87×10^{-5}	6.10×10^{-6}	9.09×10^{-6}	1.48×10^{-6}	6.02×10^{-6}	1.74×10^{-5}
6	42/C	1.5	T1 ∣ N0 ∣ II ∣ I	-	-	1.08×10^{-7}	1.36×10^{-5}	1.91×10^{-5}	3.65×10^{-7}	3.00×10^{-5}	4.99×10^{-6}
7	47/C	1.2	T1 ∣ N0 ∣ II ∣ I	-	Pre	9.58×10^{-6}	1.08×10^{-5}	7.13×10^{-6}	1.98×10^{-7}	3.24×10^{-5}	5.71×10^{-6}
8	56/C	1.5	T1 ∣ N2 ∣ II ∣ III	-	Pre	1.14×10^{-7}	9.75×10^{-6}	1.27×10^{-5}	1.65×10^{-6}	9.73×10^{-6}	1.09×10^{-5}
9	60/C	2.5	T2 ∣ N0 ∣ II ∣ II	N	Post	4.50×10^{-8}	1.61×10^{-5}	1.51×10^{-5}	1.81×10^{-6}	1.38×10^{-6}	8.57×10^{-6}
10	66/C	1.2	T1 ∣ N1 ∣ III ∣ II	-	Post	3.64×10^{-4}	7.40×10^{-6}	1.06×10^{-5}	4.13×10^{-7}	9.00×10^{-7}	4.72×10^{-6}
11	54/C	1.2	T1 ∣ N0 ∣ III ∣ I	-	-	2.69×10^{-5}	2.79×10^{-5}	2.39×10^{-5}	8.12×10^{-6}	7.95×10^{-5}	3.73×10^{-5}
12	60/C	2.0	T1 ∣ N1 ∣ II ∣ II	N	Post	1.01×10^{-3}	3.67×10^{-6}	6.96×10^{-6}	8.89×10^{-7}	1.31×10^{-4}	7.35×10^{-6}
13	58/C	1.5	T1 ∣ N0 ∣ II ∣ I	-	Post	4.72×10^{-6}	3.03×10^{-5}	1.46×10^{-5}	1.98×10^{-6}	8.53×10^{-5}	7.36×10^{-6}
14	27/C	1.5	T1 ∣ N0 ∣ III ∣ I	-	Pre	2.15×10^{-4}	1.27×10^{-1}	1.10×10^{-5}	3.05×10^{-6}	7.28×10^{-5}	1.62×10^{-5}
15	60/C	2.5	T2 ∣ N0 ∣ II ∣ II	N	Post	4.15×10^{-2}	7.34×10^{-6}	1.26×10^{-5}	1.97×10^{-6}	8.51×10^{-5}	8.83×10^{-5}
16	77/C	2.5	T2 ∣ N1 ∣ III ∣ II	N	Post	2.55×10^{-7}	7.56×10^{-6}	1.59×10^{-6}	1.81×10^{-5}	4.14×10^{-5}	4.36×10^{-5}
17	50/C	2.7	T2 ∣ N0 ∣ II ∣ II	N	Post	1.14×10^{-3}	4.72×10^{-6}	1.78×10^{-5}	2.13×10^{-6}	8.57×10^{-5}	9.79×10^{-6}
18	50/C	1.4	T1 ∣ N0 ∣ II ∣ I	-	Pre	2.58×10^{-5}	1.09×10^{-5}	1.72×10^{-5}	1.67×10^{-6}	1.67×10^{-5}	7.38×10^{-6}
19	66/C	5.0	T2 ∣ N0 ∣ II ∣ II	N	Post	2.17×10^{-8}	2.04×10^{-1}	1.10×10^{-5}	1.54×10^{-6}	8.86×10^{-6}	8.17×10^{-6}
20	49/C	4.7	T2 ∣ N2 ∣ II ∣ III	Y	Pre	2.88×10^{-3}	4.68×10^{-6}	3.57×10^{-5}	2.96×10^{-6}	2.19×10^{-4}	1.17×10^{-5}
	**Mean ± SD**					**Geometric Mean MF**					
	**55.8 ± 13.3**					**1.32×10^{-5}**	**3.66×10^{-5}**	**1.79×10^{-5}**	**1.80×10^{-6}**	**2.09×10^{-5}**	**1.19×10^{-5}**

Table S1B. *PIK3CA, KRAS, HRAS,* and *BRAF* mutant fraction measurements in HR+/HER2- ductal carcinomas

						Mutant Fraction					
		Max.	T Stage ∣ N Stage ∣			*PIK3CA*	*PIK3CA*	*KRAS*	*KRAS*	*HRAS*	*BRAF*
ID	Age/Race	Tumor Dim.	Hist. Grade ∣ Clinical Stage	Smoker	MP	H1047R	E545K	G12D	G12V	G12D	V600E
21	58/AA	15.7	T3 ∣ N3 ∣ II ∣ III	Y	-	1.55×10^{-7}	4.91×10^{-6}	8.32×10^{-6}	2.76×10^{-7}	3.66×10^{-5}	7.46×10^{-4}
22	63/C	1.9	T1 ∣ N2 ∣ II ∣ III	N	Post	9.45×10^{-8}	1.92×10^{-6}	3.55×10^{-6}	2.29×10^{-7}	3.16×10^{-5}	5.01×10^{-5}
23	65/C	5.8	T3 ∣ N2 ∣ II ∣ III	Y	Post	6.92×10^{-3}	6.71×10^{-5}	3.50×10^{-6}	1.19×10^{-7}	4.15×10^{-5}	1.67×10^{-4}
24	56/C	3.9	T2 ∣ N1 ∣ II ∣ II	Y	-	3.88×10^{-3}	7.09×10^{-6}	6.62×10^{-5}	2.36×10^{-6}	4.21×10^{-5}	1.85×10^{-5}
25	61/C	2.7	T2 ∣ N0 ∣ III ∣ II	N	Post	2.80×10^{-7}	1.14×10^{-5}	8.17×10^{-6}	6.53×10^{-7}	4.11×10^{-5}	8.19×10^{-5}
26	67/C	4.0	T2 ∣ N3 ∣ III ∣ III	N	Post	8.74×10^{-5}	5.79×10^{-6}	4.71×10^{-6}	1.45×10^{-7}	6.03×10^{-5}	5.32×10^{-5}
27	70/C	2.5	T2 ∣ N1 ∣ III ∣ II	N	-	2.53×10^{-5}	3.00×10^{-3}	1.31×10^{-5}	1.48×10^{-7}	2.26×10^{-5}	1.85×10^{-5}
28	30/C	2.2	T2 ∣ N1 ∣ I ∣ II	Y	Pre	3.85×10^{-8}	3.61×10^{-3}	5.31×10^{-6}	3.43×10^{-7}	5.68×10^{-5}	3.49×10^{-5}
29	57/AA	3.5	T2 ∣ N0 ∣ III ∣ II	N	Post	2.06×10^{-7}	1.87×10^{-5}	9.07×10^{-6}	5.50×10^{-7}	1.29×10^{-5}	9.20×10^{-5}
30	68/C	3.2	T2 ∣ N0 ∣ III ∣ II	Y	Post	2.29×10^{-6}	1.56×10^{-6}	1.66×10^{-6}	1.02×10^{-7}	2.32×10^{-5}	4.45×10^{-5}
31	69/S.Am.	4.5	T2 ∣ N2 ∣ II ∣ III	N	Post	1.38×10^{-6}	6.60×10^{-6}	3.01×10^{-6}	3.28×10^{-5}	3.87×10^{-5}	7.41×10^{-5}
32	49/?	4.5	T2 ∣ N3 ∣ I ∣ III	Y	Pre	2.06×10^{-7}	4.52×10^{-7}	2.01×10^{-5}	5.28×10^{-7}	6.15×10^{-4}	2.46×10^{-5}
33	55/C	3.5	T2 ∣ N0 ∣ III ∣ II	N	Post	6.65×10^{-2}	5.07×10^{-6}	2.57×10^{-6}	4.68×10^{-8}	1.69×10^{-5}	1.90×10^{-5}
34	53/C	4.8	T2 ∣ N0 ∣ I ∣ II	N	Post	3.17×10^{-2}	2.48×10^{-3}	1.29×10^{-5}	2.95×10^{-8}	1.68×10^{-5}	2.32×10^{-5}
35	42/C	3.2	T2 ∣ N2 ∣ III ∣ III	N	-	1.82×10^{-4}	4.95×10^{-7}	4.70×10^{-6}	1.26×10^{-7}	8.27×10^{-5}	3.18×10^{-5}
36	28/AA	2.7	T2 ∣ N0 ∣ I ∣ II	N	Pre	5.11×10^{-8}	6.82×10^{-6}	6.04×10^{-6}	6.21×10^{-7}	1.10×10^{-5}	1.80×10^{-5}
37	65/C	2.5	T2 ∣ N0 ∣ II ∣ II	N	Post	7.43×10^{-4}	5.35×10^{-6}	7.22×10^{-6}	6.39×10^{-8}	1.25×10^{-5}	2.58×10^{-4}
38	86/AA	10.5	T3 ∣ N2 ∣ - ∣ III	-	Post	6.20×10^{-8}	4.66×10^{-6}	9.06×10^{-6}	3.96×10^{-8}	1.29×10^{-5}	5.33×10^{-5}
39	49/C	4.2	T2 ∣ N0 ∣ II ∣ II	N	Pre	2.40×10^{-4}	1.56×10^{-1}	3.30×10^{-5}	5.59×10^{-7}	6.10×10^{-5}	7.36×10^{-5}
40	38/C	1.4	T3 ∣ N3 ∣ II ∣ III	Y	-	1.42×10^{-5}	3.35×10^{-5}	7.81×10^{-6}	3.89×10^{-6}	1.11×10^{-4}	7.91×10^{-6}
	**Mean ± SD**					**Geometric Mean MF**					
	**56.5 ± 14.2**					**1.20×10^{-5}**	**2.29×10^{-5}**	**7.43×10^{-6}**	**3.05×10^{-7}**	**3.59×10^{-5}**	**4.84×10^{-5}**

Table S1C. *PIK3CA, KRAS, HRAS,* and *BRAF* mutant fraction measurements in HR-/HER2+ ductal carcinomas

						Mutant Fraction					
		Max. Tumor	T Stage ∣ N Stage ∣			*PIK3CA*	*PIK3CA*	*KRAS*	*KRAS*	*HRAS*	*BRAF*
ID	Age/Race	Dim.	Hist. Grade ∣ Clinical Stage	Smoker	MP	H1047R	E545K	G12D	G12V	G12D	V600E
41	48/C	3.4	T2 ∣ N1 ∣ III ∣ II	N	Pre	1.54×10^{-7}	4.08×10^{-6}	5.33×10^{-6}	1.90×10^{-7}	3.18×10^{-5}	9.95×10^{-5}
42	48/C	1.9	T1 ∣ N0 ∣ III ∣ I	-	Pre	9.67×10^{-9}	2.98×10^{-6}	3.10×10^{-6}	5.51×10^{-8}	1.08×10^{-5}	1.73×10^{-5}
43	85/AA	1.8	T1 ∣ N1 ∣ III ∣ I	Y	Post	1.67×10^{-4}	3.37×10^{-6}	7.30×10^{-6}	2.08×10^{-5}	2.83×10^{-5}	3.80×10^{-5}
44	53/C	3.5	T2 ∣ N0 ∣ III ∣ II	Y	Post	6.84×10^{-2}	1.50×10^{-5}	2.56×10^{-5}	7.72×10^{-7}	5.36×10^{-5}	5.42×10^{-6}
45	60/C	1.5	T1 ∣ N1 ∣ - ∣ II	N	Post	4.29×10^{-6}	4.58×10^{-6}	8.27×10^{-5}	2.44×10^{-6}	4.44×10^{-5}	4.94×10^{-6}
46	54/C	1.6	T1 ∣ N- ∣ III ∣ I	N	Post	1.19×10^{-7}	3.54×10^{-6}	1.51×10^{-1}	2.58×10^{-4}	2.14×10^{-5}	5.45×10^{-6}
47	55/C	0.6	T1 ∣ N0 ∣ III ∣ I	-	Post	8.60×10^{-5}	2.93×10^{-6}	4.04×10^{-5}	1.81×10^{-4}	1.66×10^{-5}	8.16×10^{-6}
48	53/C	1.2	T1 ∣ N2 ∣ II ∣ III	-	Post	7.92×10^{-7}	5.83×10^{-6}	1.77×10^{-5}	2.75×10^{-6}	2.04×10^{-5}	5.42×10^{-6}
49	57/C	2.4	T2 ∣ N0 ∣ III ∣ II	-	Post	5.71×10^{-8}	7.77×10^{-6}	5.12×10^{-5}	1.48×10^{-7}	1.29×10^{-5}	5.93×10^{-6}
50	43/C	1.2	T1 ∣ N0 ∣ - ∣ I	-	Post	2.28×10^{-6}	4.19×10^{-6}	5.05×10^{-4}	2.78×10^{-6}	1.17×10^{-5}	5.26×10^{-6}
51	62/C	2.5	T2 ∣ N1 ∣ III ∣ II	N	Post	5.94×10^{-8}	3.92×10^{-6}	2.36×10^{-5}	4.56×10^{-5}	2.46×10^{-5}	6.34×10^{-6}
52	55/C	1.7	T1 ∣ N0 ∣ II ∣ I	Y	Post	8.32×10^{-4}	7.23×10^{-6}	6.80×10^{-5}	3.93×10^{-6}	1.48×10^{-5}	1.54×10^{-5}
53	50/C	2.5	T2 ∣ N1 ∣ III ∣ II	-	Post	7.60×10^{-8}	4.46×10^{-6}	5.17×10^{-5}	1.98×10^{-6}	9.17×10^{-5}	1.56×10^{-5}
54	51/C	2.2	T2 ∣ N0 ∣ III ∣ II	N	Post	3.80×10^{-5}	7.02×10^{-6}	1.68×10^{-5}	1.27×10^{-6}	6.24×10^{-5}	8.00×10^{-5}
55	44/C	2.5	T2 ∣ N2 ∣ III ∣ III	N	Pre	2.37×10^{-6}	1.34×10^{-1}	1.07×10^{-5}	3.89×10^{-7}	4.11×10^{-6}	8.63×10^{-6}
56	72/C	1.7	T1 ∣ N0 ∣ II ∣ I	N	Post	3.80×10^{-2}	1.66×10^{-7}	4.36×10^{-5}	5.02×10^{-6}	2.38×10^{-6}	1.16×10^{-5}
57	71/C	3.0	T2 ∣ N2 ∣ III ∣ III	N	Post	2.36×10^{-6}	5.52×10^{-6}	1.68×10^{-5}	3.19×10^{-5}	7.84×10^{-5}	7.62×10^{-6}
58	67/C	2.2	T2 ∣ N2 ∣ III ∣ III	N	Post	6.75×10^{-2}	6.45×10^{-6}	1.57×10^{-5}	1.04×10^{-4}	8.54×10^{-5}	7.51×10^{-6}
59	78/C	8.0	T4 ∣ N0 ∣ III ∣ III	-	Post	7.98×10^{-2}	3.87×10^{-5}	1.73×10^{-5}	2.25×10^{-6}	7.50×10^{-5}	7.01×10^{-5}
60	48/C	3.2	T2 ∣ N1 ∣ - ∣ II	-	Pre	1.75×10^{-5}	6.00×10^{-6}	1.19×10^{-5}	1.58×10^{-6}	1.67×10^{-4}	7.78×10^{-6}
61	69/C	4.0	T2 ∣ N0 ∣ III ∣ II	Y	Post	4.20×10^{-2}	8.60×10^{-6}	8.74×10^{-6}	1.76×10^{-6}	7.15×10^{-5}	8.12×10^{-6}
	**Mean ± SD**					**Geometric Mean MF**					
	**55.2 ± 11.5**					**2.15×10^{-5}**	**7.94×10^{-6}**	**3.47×10^{-5}**	**3.57×10^{-6}**	**2.54×10^{-5}**	**1.19×10^{-5}**

Table S1D. *PIK3CA, KRAS, HRAS,* and *BRAF* mutant fraction measurements in HR+/HER2- (TNBC) ductal carcinomas

						Mutant Fraction					
		Max. Tumor	T Stage ∣ N Stage ∣			*PIK3CA*	*PIK3CA*	*KRAS*	*KRAS*	*HRAS*	*BRAF*
ID	Age/Race	Dim.	Hist. Grade ∣ Clinical Stage	Smoker	MP	H1047R	E545K	G12D	G12V	G12D	V600E
62	64/C	1.8	T1 ∣ N2 ∣ III ∣ III	-	Post	4.44×10^{-4}	4.25×10^{-5}	3.63×10^{-6}	2.75×10^{-6}	3.48×10^{-5}	3.64×10^{-5}
63	38/C	2.2	T2 ∣ N0 ∣ III ∣ II	Y	Pre	1.17×10^{-8}	7.89×10^{-6}	3.26×10^{-6}	1.86×10^{-7}	1.85×10^{-5}	2.18×10^{-3}
64	45/C	1.8	T1 ∣ N0 ∣ III ∣ I	-	-	4.31×10^{-8}	6.01×10^{-6}	3.86×10^{-6}	1.41×10^{-6}	7.83×10^{-6}	1.66×10^{-5}
65	80/?	4.2	T2 ∣ N1 ∣ II ∣ II	Y	Post	3.15×10^{-4}	4.58×10^{-2}	6.28×10^{-5}	1.31×10^{-7}	4.01×10^{-4}	1.25×10^{-4}
66	54/C	2.2	T2 ∣ N0 ∣ III ∣ II	Y	Post	7.66×10^{-8}	7.11×10^{-6}	2.90×10^{-5}	3.25×10^{-8}	1.72×10^{-5}	8.07×10^{-5}
67	70/C	2.7	T2 ∣ N1 ∣ III ∣ II	-	-	4.67×10^{-8}	9.42×10^{-6}	1.19×10^{-5}	1.30×10^{-6}	1.44×10^{-5}	1.90×10^{-5}
68	52/C	3.0	T2 ∣ N0 ∣ III ∣ II	Y	Post	1.05×10^{-6}	6.49×10^{-6}	8.33×10^{-6}	1.70×10^{-7}	1.45×10^{-5}	3.96×10^{-5}
69	52/C	1.7	T1 ∣ N0 ∣ I ∣ I	N	Post	9.28×10^{-7}	1.13×10^{-5}	3.32×10^{-5}	1.19×10^{-6}	5.56×10^{-5}	6.10×10^{-6}
70	48/C	1.8	T1 ∣ N0 ∣ II ∣ I	N	Pre	7.94×10^{-8}	3.63×10^{-6}	1.47×10^{-5}	1.23×10^{-7}	1.12×10^{-5}	5.03×10^{-6}
71	55/C	3.0	T2 ∣ N- ∣ II ∣ II	N	Post	3.23×10^{-8}	8.05×10^{-7}	1.07×10^{-5}	5.09×10^{-8}	3.18×10^{-5}	1.05×10^{-5}
72	45/C	1.8	T1 ∣ N0 ∣ III ∣ I	-	Pre	2.40×10^{-8}	2.95×10^{-6}	1.72×10^{-5}	1.59×10^{-6}	1.98×10^{-5}	5.25×10^{-5}
73	67/C	1.5	T1 ∣ N2 ∣ III ∣ III	-	Post	1.60×10^{-4}	2.02×10^{-5}	6.37×10^{-6}	4.85×10^{-6}	6.89×10^{-5}	1.91×10^{-5}
74	40/C	1.1	T2 ∣ N0 ∣ III ∣ II	-	Pre	2.57×10^{-4}	1.52×10^{-4}	1.49×10^{-5}	4.25×10^{-6}	1.15×10^{-5}	1.01×10^{-5}
75	54/C	1.3	T1 ∣ N1 ∣ III ∣ II	-	Post	1.54×10^{-6}	5.33×10^{-4}	1.27×10^{-5}	3.25×10^{-6}	1.11×10^{-5}	1.16×10^{-5}
76	67/C	3.5	T2 ∣ N0 ∣ III ∣ II	Y	Post	1.04×10^{-7}	5.89×10^{-6}	2.03×10^{-5}	1.62×10^{-6}	3.73×10^{-5}	7.46×10^{-6}
77	74/C	2.2	T2 ∣ N0 ∣ III ∣ II	-	Post	1.29×10^{-7}	5.15×10^{-6}	1.16×10^{-5}	1.42×10^{-6}	6.33×10^{-5}	6.86×10^{-6}
78	59/His.	-	T- ∣ N- ∣ - ∣ -	-	-	1.46×10^{-6}	1.43×10^{-5}	1.60×10^{-5}	3.03×10^{-6}	2.94×10^{-5}	7.53×10^{-6}
79	49/C	3.4	T2 ∣ N0 ∣ III ∣ II	Y	-	4.16×10^{-6}	1.08×10^{-5}	7.44×10^{-6}	9.67×10^{-7}	1.35×10^{-4}	7.88×10^{-6}
80	62/C	3.8	T2 ∣ N0 ∣ III ∣ II	Y	-	6.07×10^{-7}	1.43×10^{-5}	8.95×10^{-6}	1.70×10^{-6}	5.66×10^{-5}	7.87×10^{-6}
81	43/C	4.0	T2 ∣ N0 ∣ III ∣ II	-	-	9.53×10^{-8}	7.54×10^{-6}	1.32×10^{-5}	7.51×10^{-6}	2.23×10^{-4}	7.50×10^{-6}
	**Mean ± SD**					**Geometric Mean MF**					
	**55.90 ± 11.6**					**7.64×10^{-7}**	**1.68×10^{-5}**	**1.18×10^{-5}**	**8.57×10^{-7}**	**3.34×10^{-5}**	**1.96×10^{-5}**

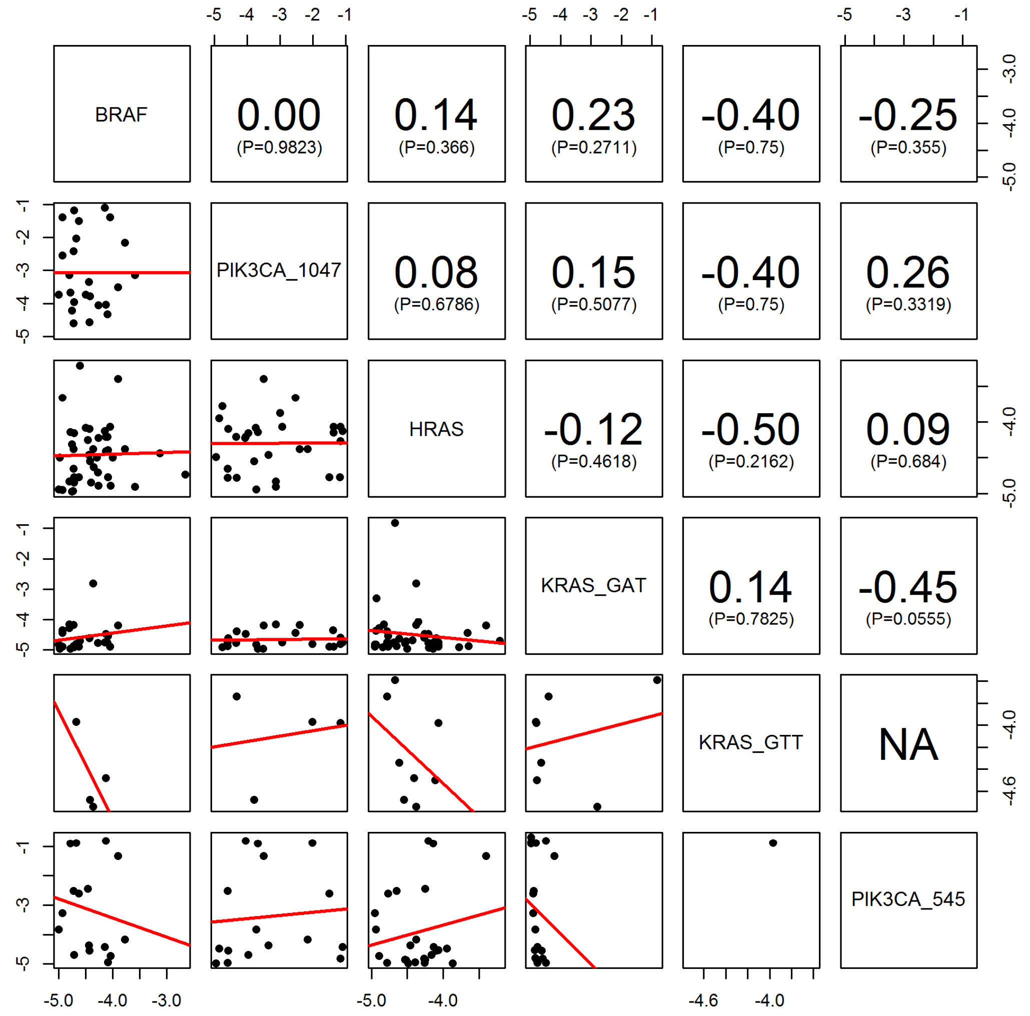

Figure S1A. Correlation matrix of MFs in ductal carcinomas: *PIK3CA* H1047R (PIK3CA_1047) and E545K (PIK3CA_545), *KRAS* G12D (KRAS_GAT) and G12V (GTT), *HRAS* G12D (HRAS), and *BRAF* V600E (BRAF).

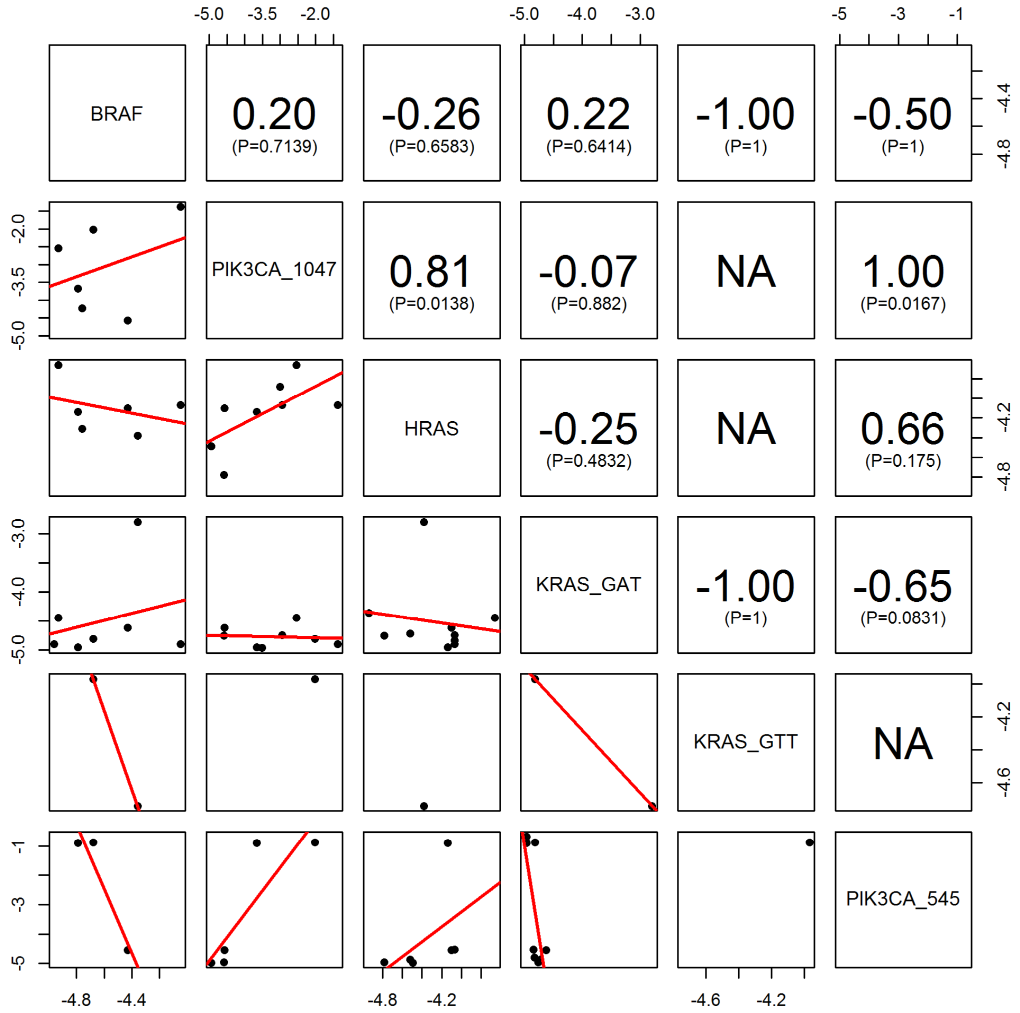

Figure S1B. Correlation matrix of MFs in HR+/HER2+ ductal carcinomas: *PIK3CA* H1047R (PIK3CA_1047) and E545K (PIK3CA_545), *KRAS* G12D (KRAS_GAT) and G12V (GTT), *HRAS* G12D (HRAS), and *BRAF* V600E (BRAF).

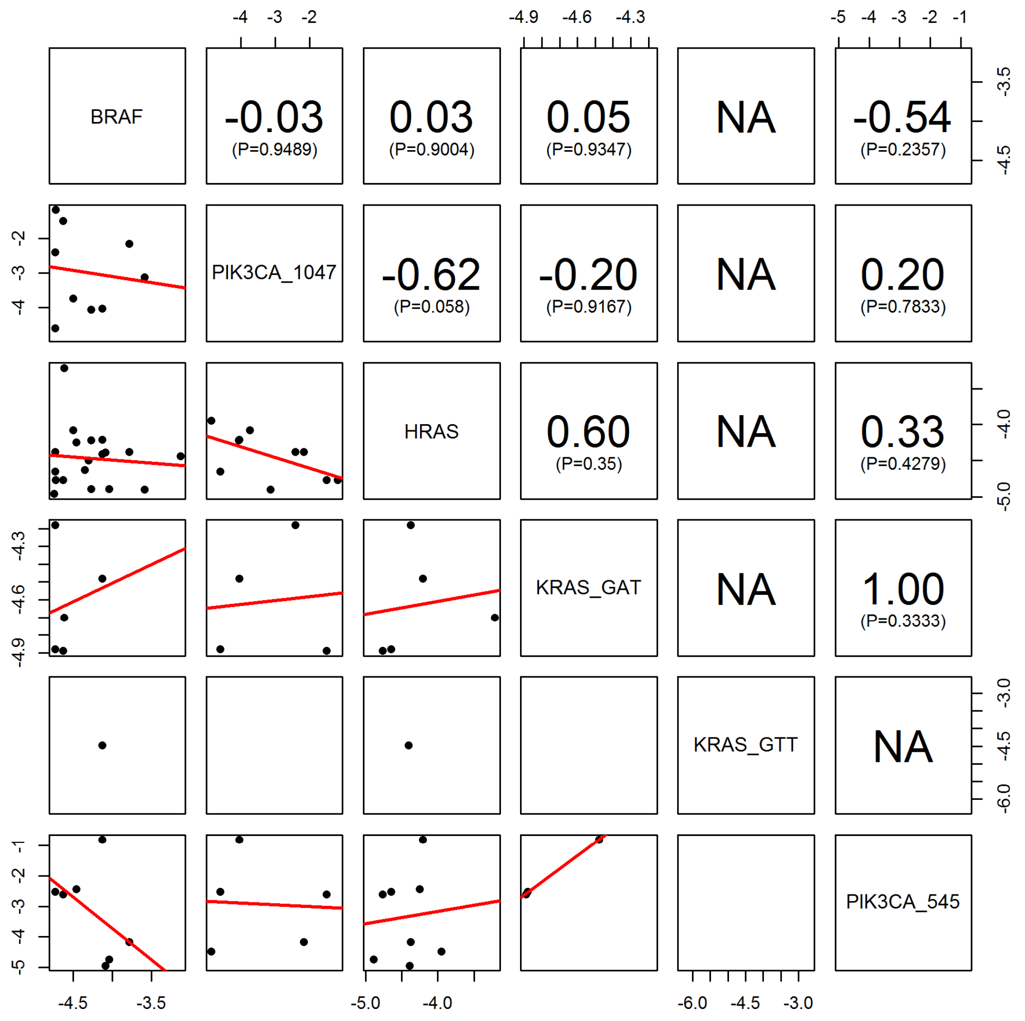

Figure S1C. Correlation matrix of MFs in HR+/HER2- ductal carcinomas: *PIK3CA* H1047R (PIK3CA_1047) and E545K (PIK3CA_545), *KRAS* G12D (KRAS_GAT) and G12V (GTT), *HRAS* G12D (HRAS), and *BRAF* V600E (BRAF).

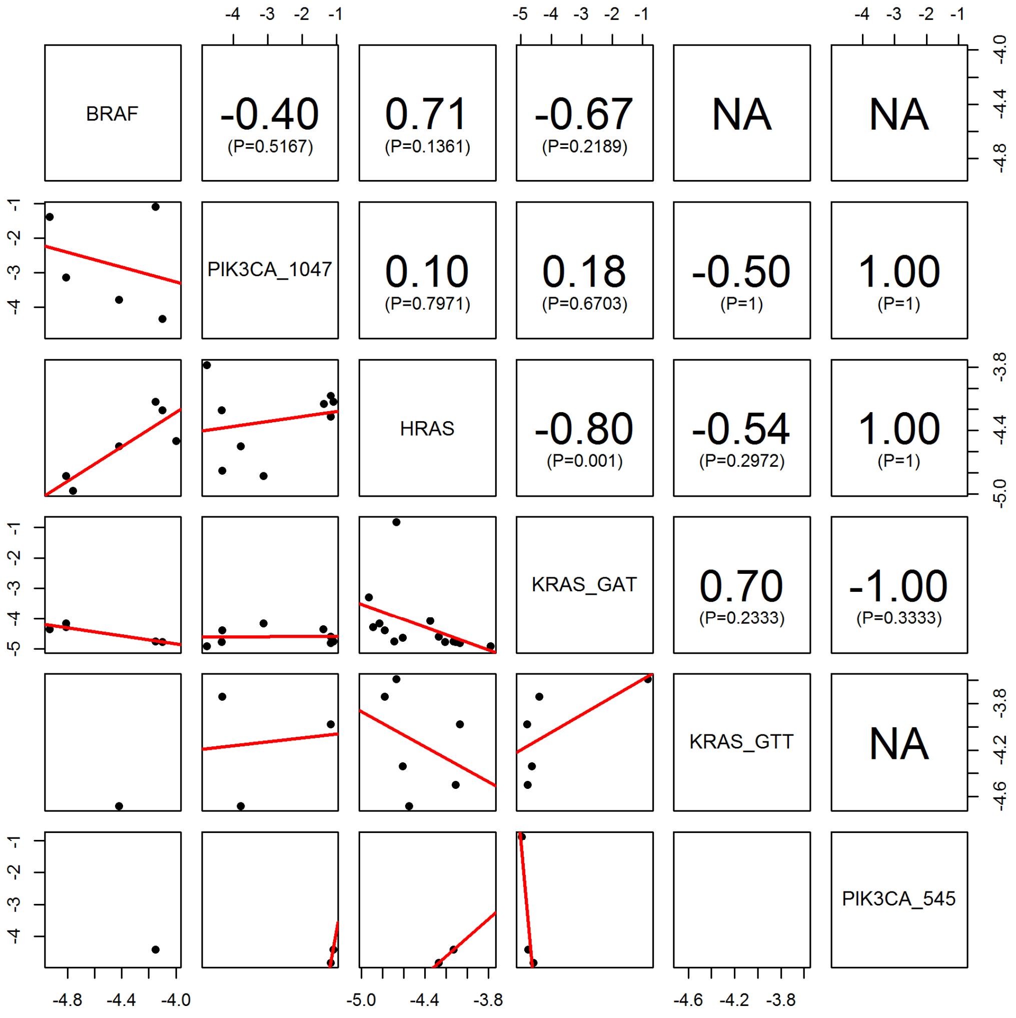

Figure S1D. Correlation matrix of MFs in HR-/HER2+ ductal carcinomas: *PIK3CA* H1047R (PIK3CA_1047) and E545K (PIK3CA_545), *KRAS* G12D (KRAS_GAT) and G12V (GTT), *HRAS* G12D (HRAS), and *BRAF* V600E (BRAF).

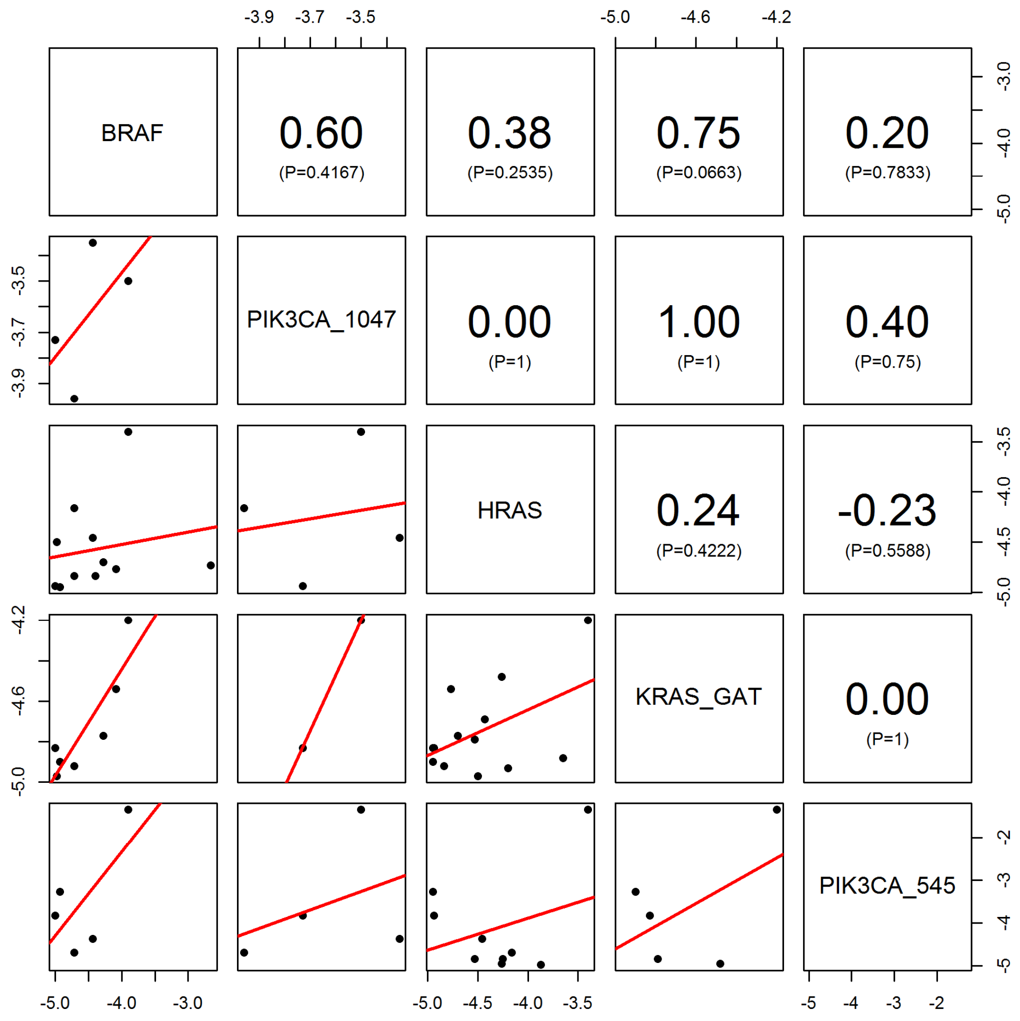

Figure S1E. Correlation matrix of MFs in HR-/HER2- (TNBC) ductal carcinomas: *PIK3CA* H1047R (PIK3CA_1047) and E545K (PIK3CA_545), *KRAS* G12D (KRAS_GAT) and G12V (GTT), *HRAS* G12D (HRAS), and *BRAF* V600E (BRAF).

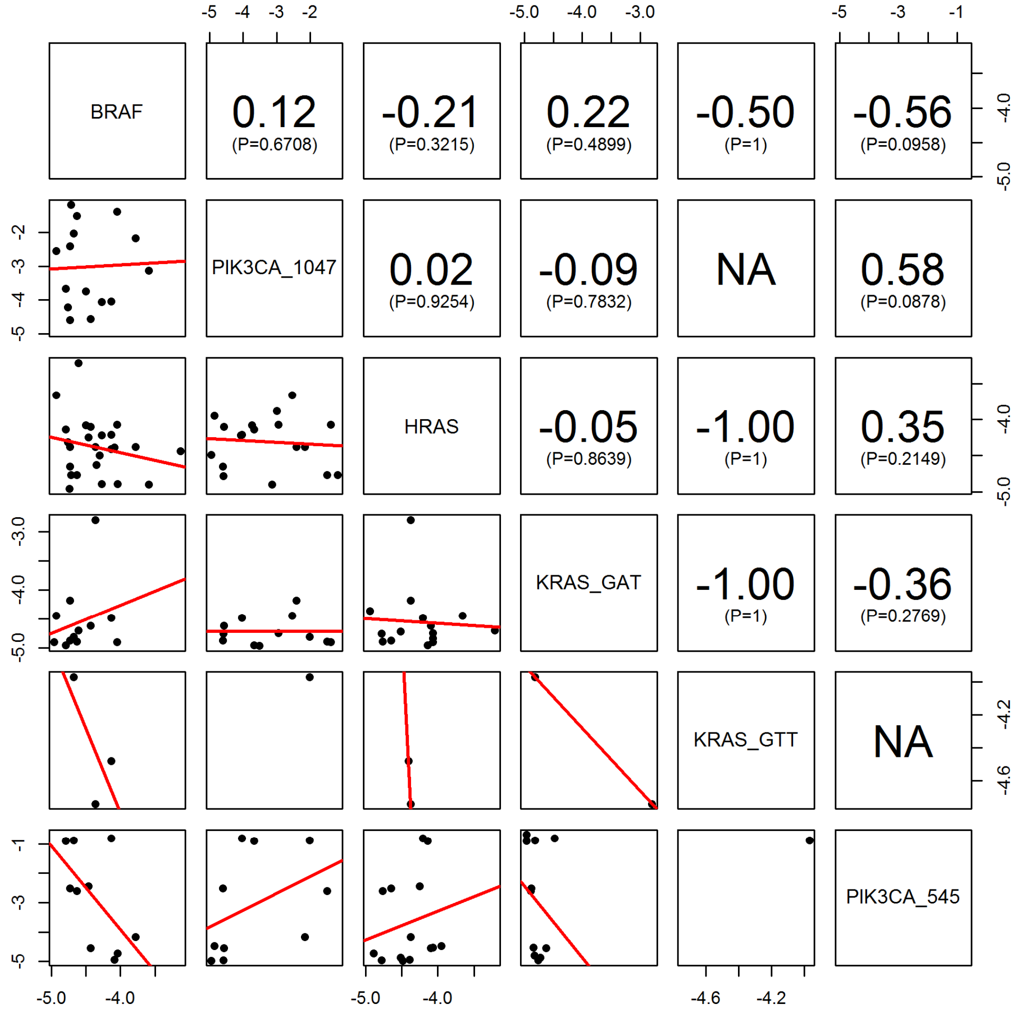

Figure S1F. Correlation matrix of MFs in ER+ ductal carcinomas: *PIK3CA* H1047R (PIK3CA_1047) and E545K (PIK3CA_545), *KRAS* G12D (KRAS_GAT) and G12V (GTT), *HRAS* G12D (HRAS), and *BRAF* V600E (BRAF).

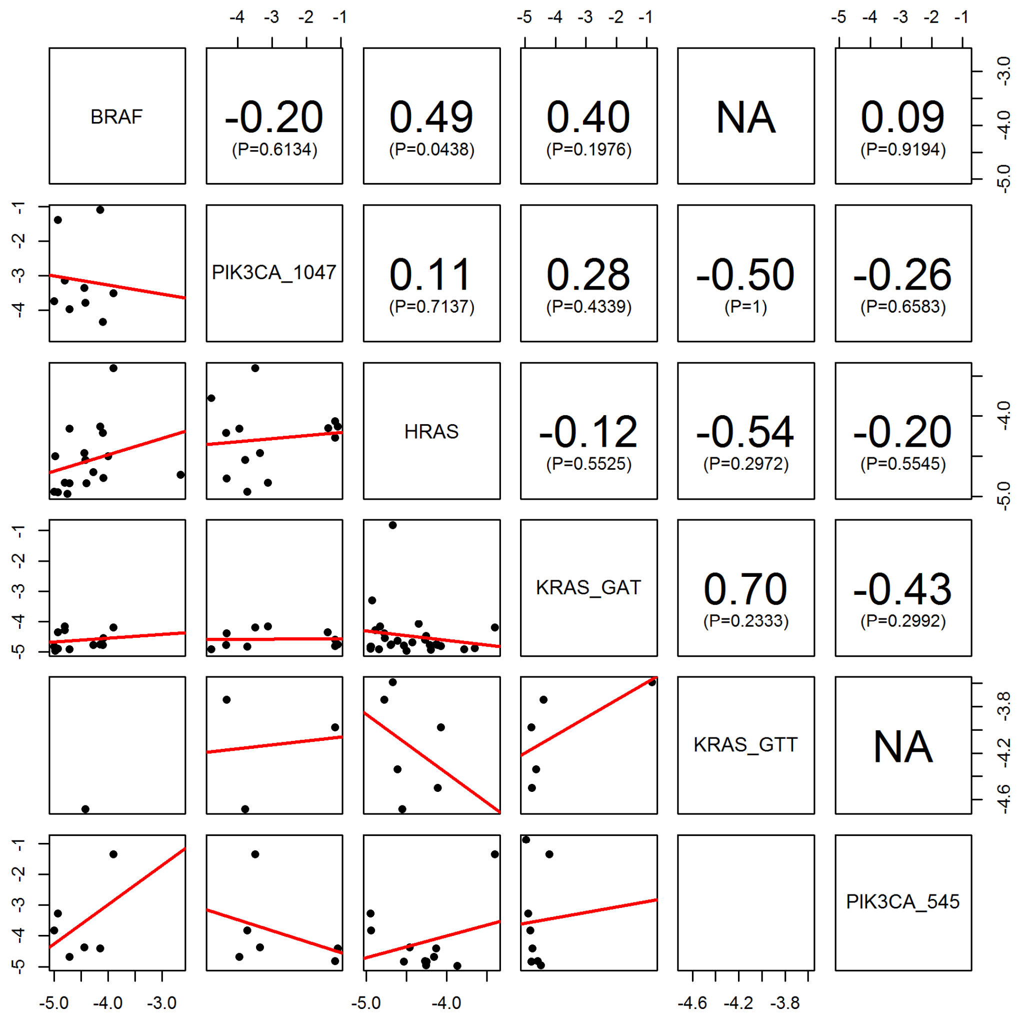

Figure S1G. Correlation matrix of MFs in ER- ductal carcinomas: *PIK3CA* H1047R (PIK3CA_1047) and E545K (PIK3CA_545), *KRAS* G12D (KRAS_GAT) and G12V (GTT), *HRAS* G12D (HRAS), and *BRAF* V600E (BRAF).

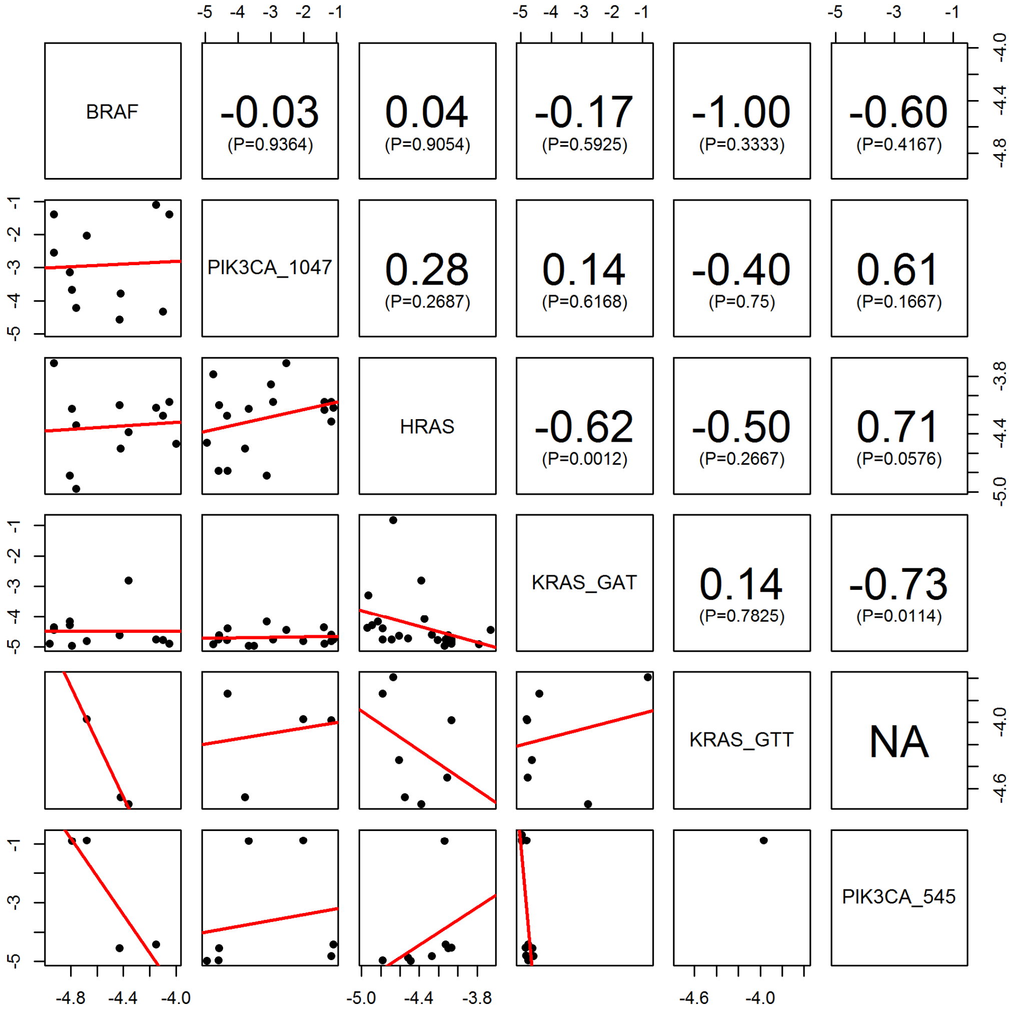

Figure S1H. Correlation matrix of MFs in HER2+ ductal carcinomas: *PIK3CA* H1047R (PIK3CA_1047) and E545K (PIK3CA_545), *KRAS* G12D (KRAS_GAT) and G12V (GTT), *HRAS* G12D (HRAS), and *BRAF* V600E (BRAF).

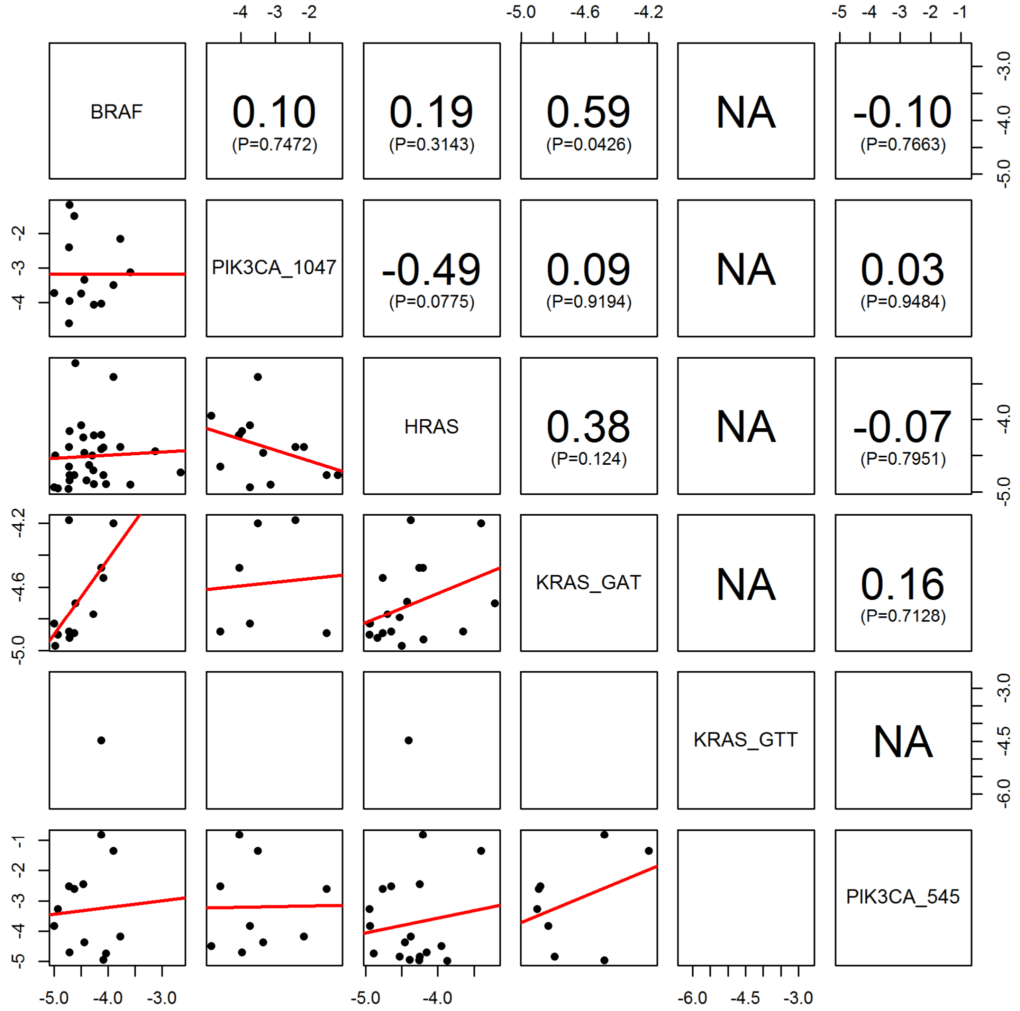

Figure S1I. Correlation matrix of MFs in HER2- ductal carcinomas: *PIK3CA* H1047R (PIK3CA_1047) and E545K (PIK3CA_545), *KRAS* G12D (KRAS_GAT) and G12V (GTT), *HRAS* G12D (HRAS), and *BRAF* V600E (BRAF).
